# Sunset Yellow protects against oxidative damage and exhibits chemoprevention in chemically induced skin cancer model

**DOI:** 10.1038/s41540-024-00349-1

**Published:** 2024-03-02

**Authors:** Saurabh Singh, Sarika Yadav, Celine Cavallo, Durgesh Mourya, Ishu Singh, Vijay Kumar, Sachin Shukla, Pallavi Shukla, Romil Chaudhary, Gyan Prakash Maurya, Ronja Lea Jennifer Müller, Lilly Rohde, Aradhana Mishra, Olaf Wolkenhauer, Shailendra Gupta, Anurag Tripathi

**Affiliations:** 1 Food Toxicology Group, CSIR- Indian Institute of Toxicology Research, 226001 Lucknow, India; 2 Academy of Scientific and Innovative Research (AcSIR), 201002 Ghaziabad, India; 3 University of Strasbourg, F-67081 Strasbourg, France; 4 Department of Systems Biology and Bioinformatics, University of Rostock, 18055 Rostock, Germany; 5 Drug and Chemical Toxicology Group (FEST), CSIR- Indian Institute of Toxicology Research, 226001 Lucknow, India; 6 Division of Microbial Technology, CSIR-National Botanical Research Institute, 226001 Lucknow, India; 7 Center for Advanced Studies, Dr APJ Abdul Kalam Technical University, 226031 Lucknow, India; 8 Leibniz-Institute for Food Systems Biology at the Technical University of Munich, 85354 Freising, Germany; 9 Chhattisgarh Swami Vivekananda Technical University, 491107 Bhilai, India

**Keywords:** Target identification, Target identification, Cancer

## Abstract

Skin cancer and other skin-related inflammatory pathologies are rising due to heightened exposure to environmental pollutants and carcinogens. In this context, natural products and repurposed compounds hold promise as novel therapeutic and preventive agents. Strengthening the skin’s antioxidant defense mechanisms is pivotal in neutralizing reactive oxygen species (ROS) and mitigating oxidative stress. Sunset Yellow (SY) exhibits immunomodulatory characteristics, evidenced by its capacity to partially inhibit the secretion of proinflammatory cytokines, regulate immune cell populations, and modulate the activation of lymphocytes. This study aimed to investigate the antioxidant and anti-genotoxic properties of SY using in-silico, in vitro, and physiochemical test systems, and to further explore its potential role in 7,12-dimethylbenz(a) anthracene (DMBA)/ 12-o-tetradecanoylphorbol-13-acetate (TPA)-induced two-stage skin carcinogenesis. In vitro experiments showed that pre-treatment of SY significantly enhanced the cell viability of HaCaT cells when exposed to tertiary-Butyl Hydrogen Peroxide (tBHP). This increase was accompanied by reduced ROS levels, restoration of mitochondrial membrane potential, and notable reduction in DNA damage in (SY + tBHP) treated cells. Mechanistic investigations using DPPH chemical antioxidant activity test and potentiometric titrations confirmed SY’s antioxidant properties, with a standard reduction potential (${E}^{o}$) of 0.211 V. Remarkably, evaluating the effect of topical application of SY in DMBA/TPA-induced two-step skin carcinogenesis model revealed dose-dependent decreases in tumor latency, incidence, yield, and burden over 21-weeks. Furthermore, computational analysis and experimental validations identified GSK3β, KEAP1 and EGFR as putative molecular targets of SY. Collectively, our findings reveal that SY enhances cellular antioxidant defenses, exhibits anti-genotoxic effects, and functions as a promising chemopreventive agent.

## Introduction

Skin is the body’s largest organ, exposed to various pollutants and chemical carcinogens in our day-to-day life^1^. Environmental carcinogens, UV rays, and inflammatory agents have been known to promote skin-related pathologies and cancers^2–4^. Skin cancer is rapidly growing across the globe due to heightened exposure to anthropogenic environmental pollutants and UV rays^5^. It is well established that the toxic effects of diverse environmental pollutants are mediated by reactive oxygen species (ROS), causing redox imbalance, oxidative stress, and genotoxicity^4,6–9^. Oxidative stress arises when the generation of reactive oxygen species (ROS) surpasses the body’s endogenous antioxidant defense mechanisms and enhanced exposure to exogenous antioxidants can effectively neutralize ROS^10^. Previous experimental studies have demonstrated that antioxidants can mitigate oxidative damage and potentially prevent the development of human diseases, including cancer^11^. These evidences suggest that topical application of antioxidants can suppress the level of ROS, thereby reducing the risk of skin-related inflammatory pathologies and cancer. Identifying natural products and repurposed compounds provides an opportunity to develop new preventive strategies and treatments for skin-related pathologies and cancers. One promising compound is Sunset Yellow, which is investigated in the study.

SY is a synthetic permitted food color used in many countries across the globe. It is added to various food products like sweets, ice candy, cosmetics, and beverages and also as a medicine excipient^12^. In vitro and in vivo investigations with SY didn’t show any kind of mutagenic and carcinogenic effects^13,14^. Other toxicological studies also found no significant toxic manifestations after acute, chronic, and long-term exposure^12^. Based on the study endpoints, the NOAEL (No Observed Adverse Effect Level) of SY was observed to be 375 mg/kg b.wt., and JECFA (Joint FAO/WHO Expert Committee on Food Additives) fixed an ADI (Acceptable Daily Intake) of 0–4 mg/kg b.wt. in humans^15^. Few studies have also documented that SY could have anti-inflammatory properties. Hashem et al.^16^ reported that SY reduced the monocyte counts and lowered the delayed-type hypersensitivity response in animal model^16^. In a study conducted in our laboratory, we also observed that SY partially suppressed the lymphocyte proliferation and reduced the levels of proinflammatory cytokines IL-2, IL-6, IL-4, IL-17, TNFα, and IFN-_ϒ_ in mitogen-activated splenocytes^12^. Classically, it has been seen that chemo-therapeutic cancer drugs also interfere with the proliferation of lymphocytes^17^; however, their effects are more profound in magnitude, causing undesirable systemic immunosuppression in patients. Notably, SY is a permitted food color and in the long history of exposure to humans, SY has not been found to cause immunosuppression or other target organs toxicity^1^. In addition, anti-inflammatory agents may also reduce the levels of ROS^12^, which are implicated in the development of cancer and other inflammatory pathologies^18,19^.

The current study was designed to test and characterize the protective effect of SY against oxidative damage induced by tBHP in human keratinocyte-derived HaCaT cells. It is well established that tBHP causes oxidative stress and cellular damages by depleting the cellular antioxidant levels and formation of hydroxyl radicals^20,21^. The antioxidant potential of SY was then investigated by measuring the standard potentiometric reduction potential (E°) and by determining the chemical antioxidant activity of the compound. Furthermore, to study the implications of SY’s antioxidant properties observed in our in vitro and physiochemical investigations, we evaluated the chemopreventive effects of SY topical application in DMBA/TPA-induced three-stage skin cancer models. In this well-established chemical-induced skin cancer model, DMBA causes DNA modification in the first step, followed by TPA, causing inflammation and proliferation^22^. Further, we speculated that in addition to anti-oxidant activity of SY, the effect of SY on signaling pathways may also play an additional co-operative role in prevention of skin carcinogenesis. Therefore, in silico studies were carried out to identify the potential molecular targets of SY in reference to its antioxidant and chemopreventive properties. Finally, the putative targets of SY were also validated through immunoblotting.

## Results

### SY protects against tBHP-induced cell death in HaCaT cells

**Fig. 1:**
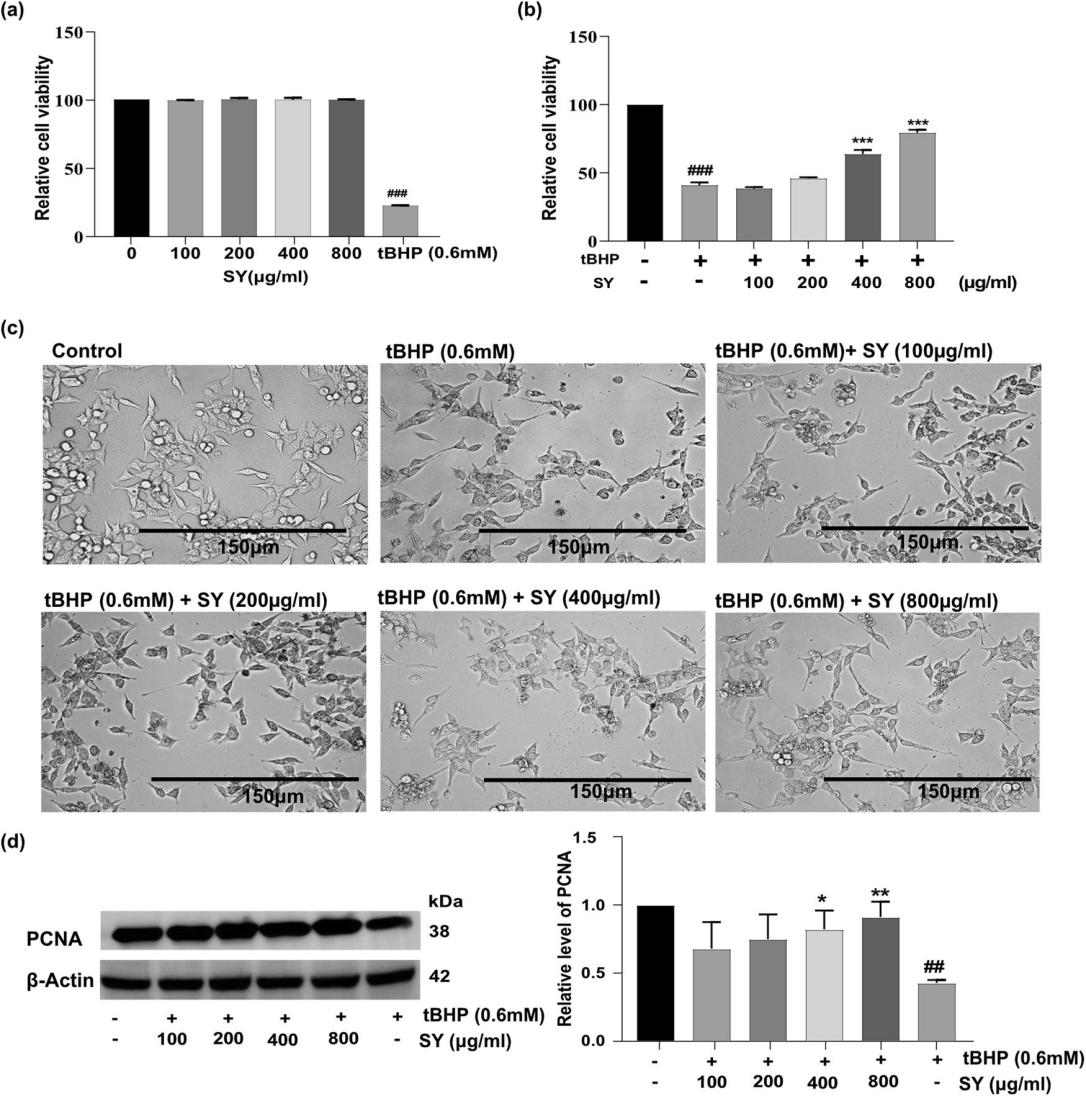
Sunset Yellow (SY) protects against tBHP – induced cytotoxicity. **a** Viability of HaCaT cells after 24 h of treatment with various concentrations of SY. **b** Viability of HaCaT cells treated with SY (100–800 µg/ml) for 12 h, followed by treatment with 0.6 mM tBHP for 6 h. **c** Effect of pretreating HaCaT cells with varying concentrations of SY (100–800 µg/ml) on tBHP (0.6 mM)-induced cytotoxicity. Cells were seeded at a density of 2 × 10^5^ cells/well. After 24 h, cells were treated with SY for 12 h and tBHP for 6 h. **d** Effect of pretreating HaCaT cells with varying concentrations of SY (100–800 µg/ml) on tBHP (0.6 mM)-induced PCNA protein expression. The protein levels were assessed by western blotting and quantified by densitometric analysis (*n* = 3). Results are presented as Mean ± standard error, combining data from three independent experiments. The differences among the groups were analyzed with one-way ANOVA followed by the Tukey post hoc test. ^###^*P* < 0.001, ^##^*P* < 0.01 vs. untreated control; *P < 0.05, **P < 0.01, ***P < 0.001 vs. tBHP treated cells.

To investigate the protective effects of SY against tBHP-induced cytotoxicity in HaCaT cells, firstly, we evaluated the effect of different concentrations of SY (100 µg/ml to 800 µg/ml) on HaCaT cells over a 24-hour period, using PI (Propidium Iodide) staining to analyze cell viability (Fig. 1a). Our results indicated that SY did not exhibit any significant cytotoxic effects on HaCaT cells during this time period. Next, we examined the effects of SY on tBHP-induced cytotoxicity in HaCaT cells. We pretreated HaCaT cells with various concentrations of SY (100 µg/ml to 800 µg/ml), followed by exposing the cells to a cytotoxic concentration of tBHP (0.6 mM). The group of cells treated with tBHP alone demonstrated a decrease of over 50% in cell viability compared to the control group. However, when SY was administered, it reversed the cytotoxic effects induced by tBHP through oxidative stress in a dose-dependent manner (Fig. 1b). We also carried out a morphological analysis of different treatment groups. Clearly, tBHP-treated cells showed cell shrinkage with a significantly deformed cellular morphology, loss of cell-cell contacts, karyopyknosis with increased cell debris; however, increasing doses of SY (100–800 µg/ml) imparted notable protection against these morphological alterations (Fig. 1c).

The active nuclear protein known as proliferating cell nuclear antigen (PCNA) is involved in the replication, recombination, and repair of DNA. The normal expression of PCNA in proliferating cells reflects healthy cellular physiology. In comparison to untreated cells, we observed that tBHP treated cells exhibited reduced PCNA protein expression. However, HaCaT cells pretreated with SY showed a dose dependent enhancement in expression of PCNA, when compared to tBHP treated cells (Fig. 1d).

### SY pretreatment effectively reduces tBHP-induced intracellular ROS generation in HaCaT cells

**Fig. 2:**
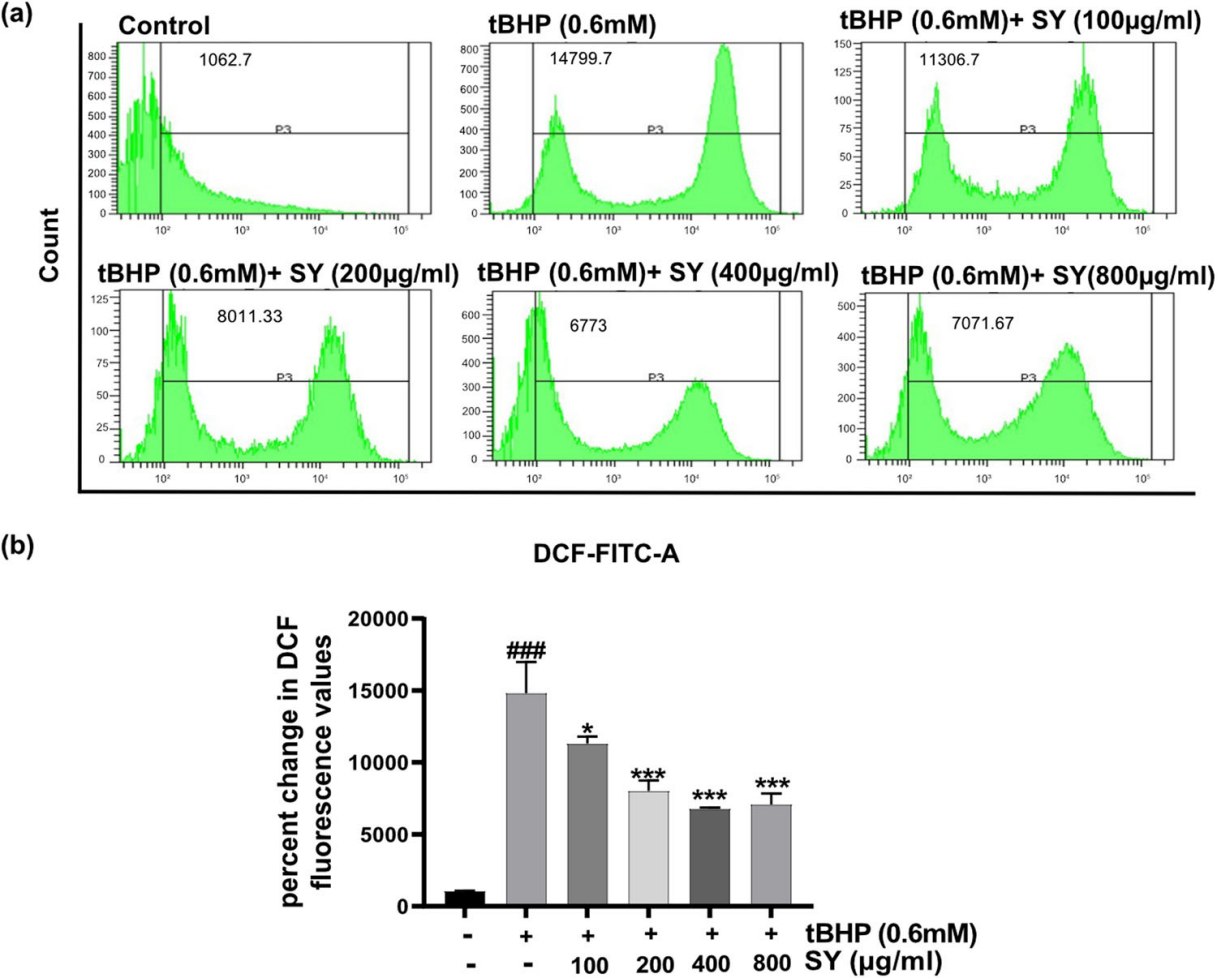
SY suppresses tBHP-induced ROS generation. **a** Flow cytometry analysis of HaCaT cells treated with various concentrations of SY for 12 h, followed by exposure to 0.6 mM tBHP for 6 h. **b** Fluorescence intensity representing ROS levels was quantified and presented as the mean ± standard error of the mean (SEM) from three independent experiments (*n* = 3). The differences among the groups were analyzed with one-way ANOVA followed by the Tukey post hoc test. ^###^*P* < 0.001 vs. untreated control group; *P < 0.05 and ***P < 0.001 vs. tBHP treated group.

Intracellular reactive oxygen species (ROS) levels were quantified in HaCaT cells pre-treated with SY and subsequently exposed to tBHP. The fluorescence probe DCFH_2_-DA (2′,7′-dichlorodihydrofluorescein-diacetate) was employed to estimate ROS levels over a temporal course. The experimental data demonstrated a significant increase in fluorescence intensity in HaCaT cells following tBHP treatment, indicative of heightened intracellular ROS generation. In contrast, pre-treatment of HaCaT cells with SY at concentrations ranging from 100 to 800 µg/ml resulted in a dose-dependent reduction of intracellular ROS generation; however, the response plateaued between 400 and 800 µg/ml. These results underscore the ability of SY to mitigate tBHP-induced oxidative stress by attenuating intracellular ROS levels in a concentration-dependent manner (Fig. 2).

### SY pretreatment protects against tBHP-induced mitochondrial dysfunction in HaCaT cells

**Fig. 3:**
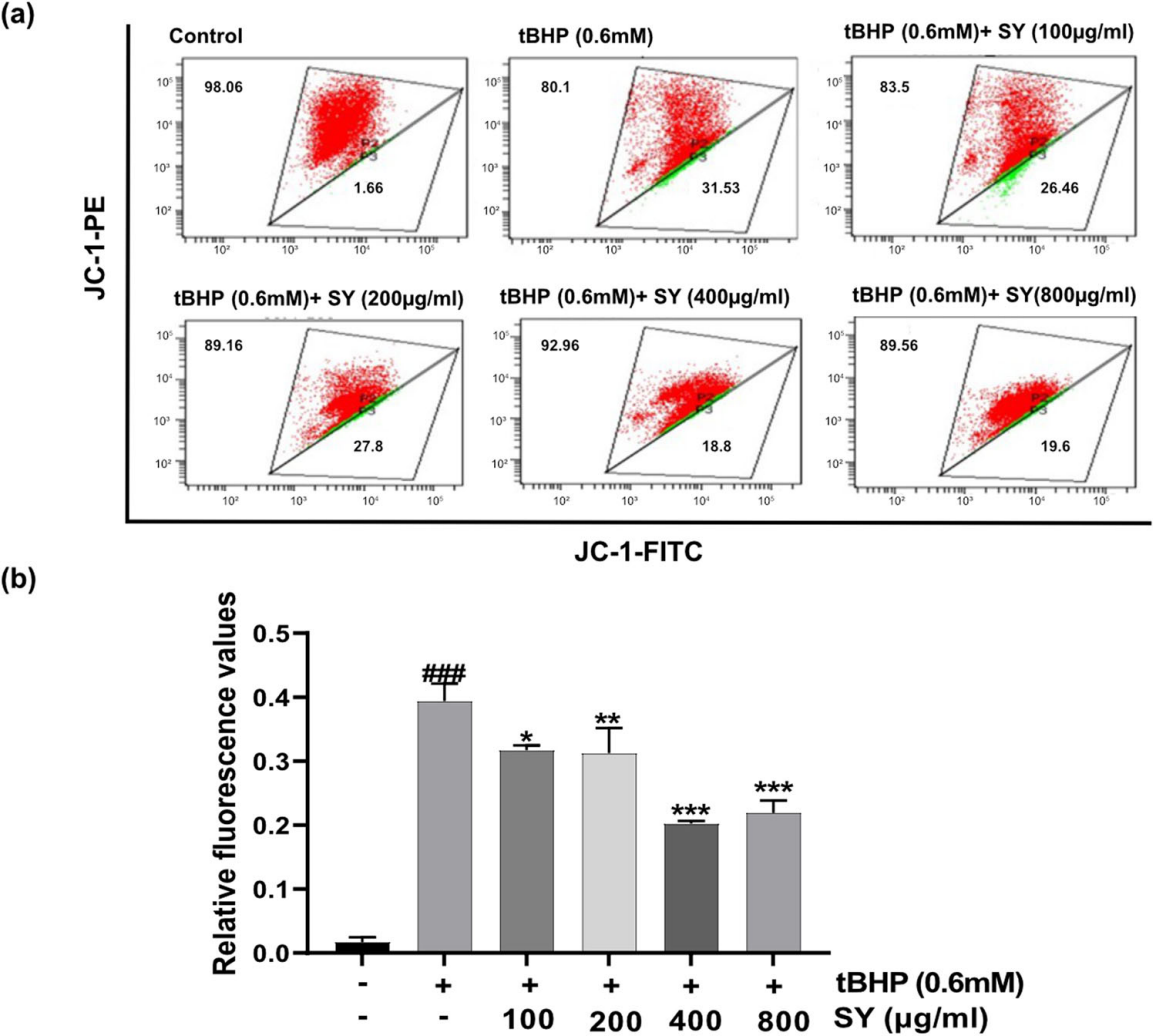
SY protects against tBHP-induced mitochondrial dysfunction. **a** Loss of mitochondrial membrane potential (MMP) was assessed using JC-1 staining. **b** The fluorescence intensity [measured by estimating the fluoroscence of low MMP apoptotic cells (gate-P3) with respect to high MMP healthy cells (gate-P2)] is presented as the mean ± standard error of the mean from three independent experiments (*n* = 3). The differences among the groups were analyzed with one-way ANOVA followed by the Tukey post hoc test. Statistical significance is indicated as ^###^*P* < 0.001 vs. untreated control; **P < 0.01, and ***P < 0.001 vs. tBHP treated cells.

Since enhanced ROS generation is often a consequence of mitochondrial dysfunction, therefore, we assessed the mitochondrial membrane potential (MMP) of HaCaT cells under different treatment conditions of our study. A membrane-permeable JC-1 dye which serves as an excellent marker of membrane depolarization, was used to assess the MMP alterations in order to monitor the mitochondrial dysfunction. Comparative analysis of MMP alterations between tBHP-treated HaCaT cells and SY-pretreated cells stimulated with tBHP revealed notable differences. In tBHP-treated cells, MMP exhibited a significant reduction, as evidenced by a substantial change in JC-1 (5,5′,6,6′-Tetrachloro-1,1′,3,3′-tetraethyl benzimidazolocarbocyanine iodide) fluorescence intensity (The green fluorescence signals increased and the red fluorescence signals decreased). Conversely, in SY-pretreated cells exposed to tBHP, the magnitude of MMP change was considerably lower (Faint green signals and strong red signals of JC-1 dye). This finding indicates that SY treatment conferred protection against tBHP-induced mitochondrial dysfunction in HaCaT cells, as evidenced by the attenuated alteration in MMP levels (Fig. 3).

### SY pretreatment attenuates tBHP-induced DNA damage and micronucleus formation in HaCaT cells

**Fig. 4:**
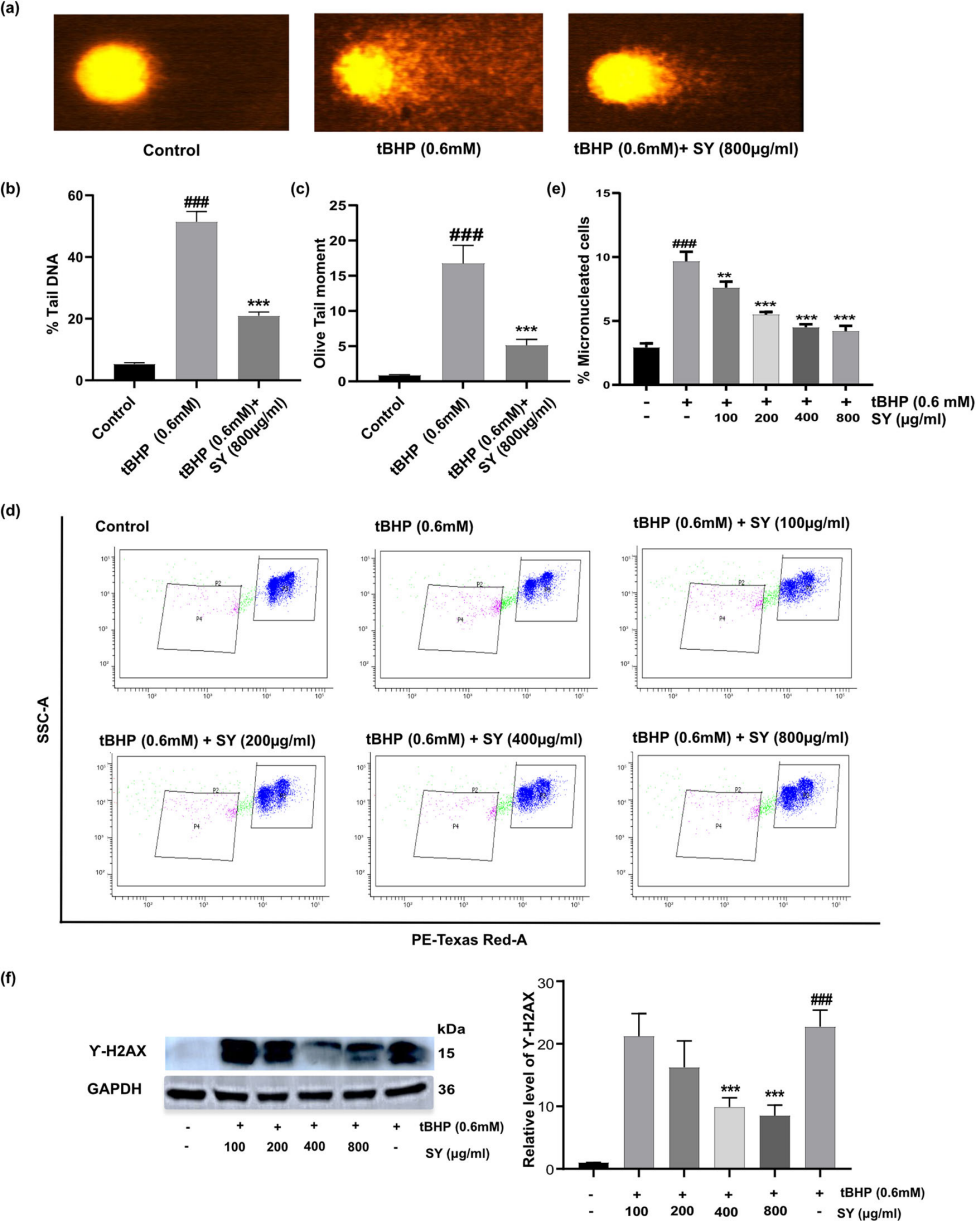
Protective effects of SY against tBHP-induced DNA damage in HaCaT cells. **a** Representative fluorescent images of neutral Comet showing control cells, tBHP-treated cells, and tBHP + SY (800 µg/ml) treated cells after pre-treatment with the indicated amount of SY for 12 h, followed by treatment with 0.6 mM tBHP for 6 h. Data showed are derived from three independent experiments (*n* = 3). **b** Percentage of Tail DNA showing the amount of DNA breaks (**c**) Olive Tail movement (OTM) measurements showing the amount of DNA breaks. **d** SY-mediated attenuation of tBHP-induced micronucleus formation in HaCaT cells, assessed by flow cytometry [measured by estimating the percentage of micronuclei (gate-P4) with respect to intact nuclei (gate-P3)]. **e** Bar graph representing micronucleus formation (*n* = 3). **f** Effect of pretreating HaCaT cells with varying concentrations of SY (100–800 µg/ml) on tBHP (0.6 mM)-induced ϒ-H2AX protein expression. The protein levels were assessed by western blotting and quantified by densitometric analysis (*n* = 3). The differences among the groups were analyzed with one-way ANOVA followed by the Tukey post hoc test. Results are presented as Mean ± standard error, combining data from three independent experiments. Statistical significance: ^###^*P* < 0.001 vs. untreated control; **P < 0.01, and ***P < 0.001 vs. tBHP-treated cells.

Since the toxic effects of excessive ROS result into damaged DNA, therefore, we also examined the effect of SY pretreatment on tBHP-induced DNA damage. To evaluate the impact of SY pretreatment on tBHP-induced DNA damage, we assessed the levels of DNA damage parameters using the alkaline comet assay. HaCaT cells were pretreated with SY for 12 h, followed by exposure to tBHP for an additional 6 h. Representative image of the comet assay showing DNA fragmentation (visualized as scattered DNA smear) in tBHP (0.6 mM) only group. Whereas SY (800 μg/ml) pretreatment clearly protected against the DNA damage induced by tBHP (Fig. 4a). The extent of DNA damage was measured by quantifying the percent Tail DNA and olive tail moment (OTM). In the tBHP + SY (800 µg/ml) group, a significant decrease in the percent Tail DNA was observed, measuring 20.91%. In contrast, the tBHP-only group exhibited a significantly higher percent Tail DNA of 51.4%. These results demonstrate that pre-treatment with SY effectively decreased the level of DNA damage caused by tBHP in HaCaT cells. Furthermore, the evaluation of OTM, another parameter of DNA damage revealed a substantial decrease in the tBHP + SY (800 µg/ml) group compared to the tBHP-only group. The OTM was measured at 16.75 in the tBHP-only group, while in the SY (800 µg/ml) + tBHP group, it was significantly lower at 5.12. In comparison, the control group exhibited a percent Tail DNA and OTM of 5.31% and 0.88, respectively (Fig. 4a, b, c). These findings demonstrate the protective effect of SY pretreatment against tBHP-induced DNA damage in HaCaT cells.

The formation of micronuclei or chromosomal fragments, which are small DNA-containing particles and a prominent marker of genotoxicity, was also examined in different treatment groups. Our data revealed a significant increase in micronuclei formation in the tBHP group compared to the control group. However, pre-treatment of cells with SY (100–800 µg/ml) followed by tBHP exposure demonstrated a remarkable decrease in micronucleus formation at all the doses of SY.

In the tBHP group, the frequency of micronuclei formation was 9.30 indicating the genotoxic effect of tBHP-induced oxidative stress. Conversely, pre-treatment with SY (100–800 µg/ml) followed by tBHP exposure led to a dose-dependent reduction in micronucleus frequency. The frequencies of micronuclei in the SY + tBHP groups at 100, 200, 400, and 800 µg/ml doses of SY were found to be 7.28, 5.38, 4.42, and 4.16, respectively (Fig. 4d, e). The significant decrease in micronucleus frequency in the SY + tBHP groups indicates the protective effect of SY against genotoxicity induced by tBHP-induced oxidative stress.

Double strand breaks are among the most serious forms of DNA damage that are difficult for normal cells to repair. Cells need to phosphorylate γ-H2AX in order to detect and initiate repair of DNA damage. γ-H2AX expression also acts as an excellent marker of DNA damage. Using western blot analysis, the phosphorylation of the γ-H2AX was also investigated (Fig. 4f). Evidently, tBHP treated HaCaT cells showed enhanced expression of γ-H2AX indicating marked DNA damage, however, tBHP + SY treated cells exhibited a SY dose dependent decrease in the expression of γ-H2AX (Fig. 4f).

### Measurement of standard reduction potential (${E}^{o}$) and DPPH activity depicting antioxidant potential of SY

**Fig. 5:**
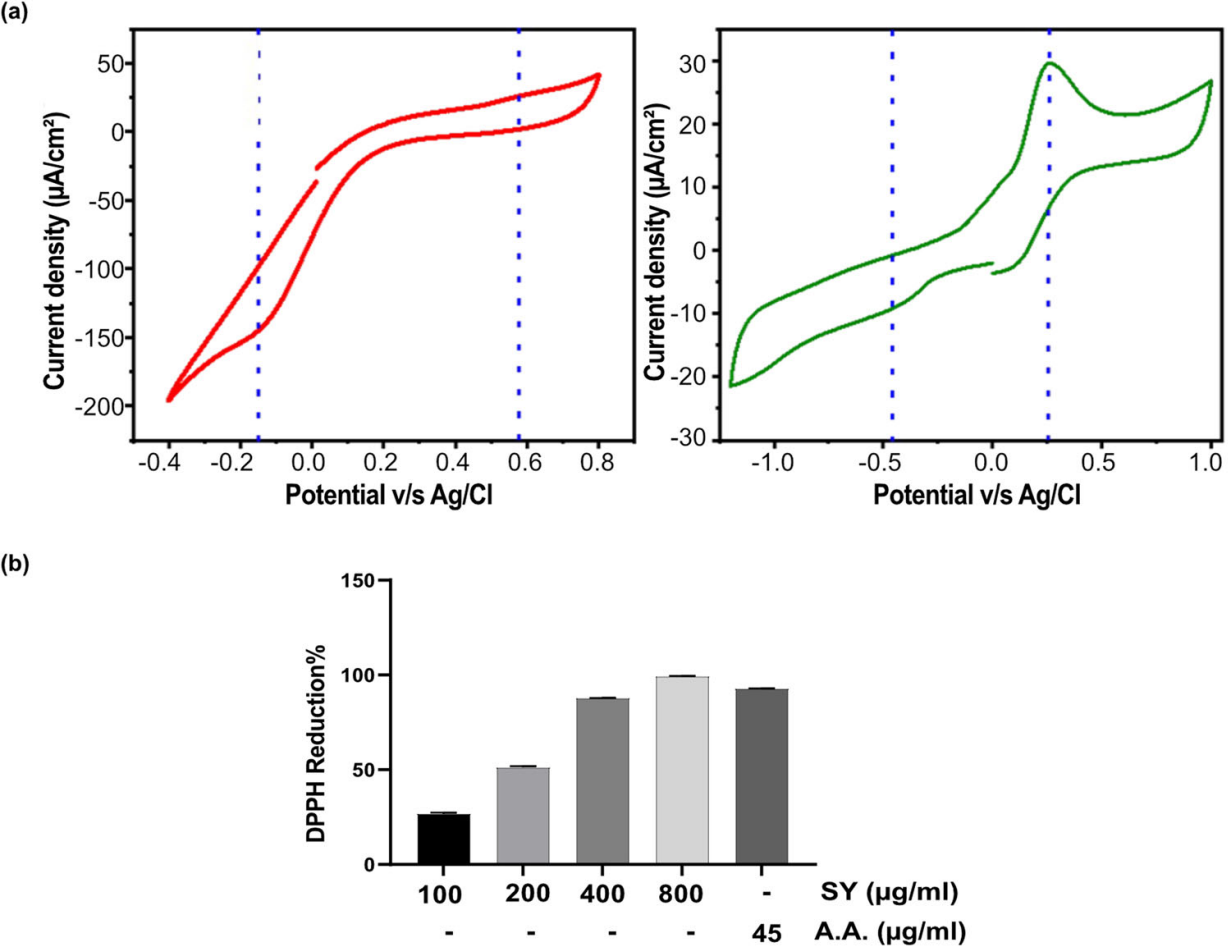
Comparison of standard reduction potential and DPPH activity of SY and Ascorbic acid. **a** Cyclic voltammetry (CV) curve for SY dye (red) for Ascorbic acid (green) (**b**) DPPH radical scavenging activity of SY (100–800 µg/ml) and Ascorbic acid (45 µg/ml). Results are presented as combining data from three independent experiments (*n* = 3).

Since SY imparts protection against oxidative stress and genotoxic damage, therefore, we measured the antioxidant activity of SY through examination of its standard reduction potential (${E}^{o}$) and DPPH (2,2-diphenyl-1-picrylhydrazyl) activity. The antioxidant potential was also compared with the well-known antioxidant ascorbic acid (Vitamin C). Figure 5a depicts the cyclic voltammograms for SY dye and ascorbic acid, respectively, enabling the estimation of their reduction potentials. As shown in Fig. 5a, the oxidation and reduction peaks of SY dye were observed at approximately 0.577 V and −0.15 V, respectively. Similarly, the oxidation and reduction peaks of ascorbic acid were found at around 0.255 V and −0.457 V, respectively.

The standard redox potential from oxidation and reduction peak potential is given by the Eq. 1^23^

1 $${E}^{o}=\frac{{E}_{{op}}+{E}_{{rp}}}{2}$$

Where, ${E}_{{op}}$ is the oxidation potential; ${E}_{{rp}}$ is the reduction potential, and ${E}^{o}$ is the standard reduction potential.

Table 1 presents the standard reduction potentials of SY and ascorbic acid. Although ascorbic acid, a well-known antioxidant, exhibited a lower negative potential (indicating better antioxidant activity) of −0.1 V compared to SY, the values of their standard reduction potentials are relatively close. The close proximity of the standard reduction potentials of SY and ascorbic acid further supports the notion that SY possesses promising antioxidant properties.

**Table 1:** Cyclic voltammetric oxidation and reduction peaks for SY dye and Ascorbic acid

Electrolyte	Oxidation potential (${E}_{{op}}$)	Reduction Potential (${E}_{{rp}}$)	Standard reduction Potential (${E}^{o}$)
SY DYE	0.577	−0.150	0.211
Ascorbic Acid	0.255	−0.457	−0.100

Next, the scavenging activity of SY and ascorbic acid against DPPH free radicals was evaluated using the DPPH (2,2-diphenyl-1-picrylhydrazyl) assay. SY exhibited remarkable DPPH scavenging activity, as evidenced by the significant inhibition of DPPH free radicals at different concentrations (100–800 µg/mL). Notably, the DPPH scavenging activity of SY also showed a dose-dependent relationship. At concentrations of 100, 200, 400, and 800 µg/mL, SY demonstrated substantial inhibition of DPPH free radicals, with percentages of 26.65%, 51.16%, 87.74%, and 99.26%, respectively. In comparison, ascorbic acid (at a concentration of 45 µg/mL) exhibited a scavenging activity of approximately 92.83% (Fig. 5b).

These findings highlight the potent DPPH scavenging activity of SY and suggests its potential as an effective antioxidant agent. Furthermore, the scavenging activity of SY was observed to be comparable to that of Ascorbic acid, a well-known antioxidant. These results underscore the promising antioxidant properties of SY and support its potential applications in combating oxidative stress-related conditions.

### A two-stage skin carcinogenesis model demonstrates SY’s chemopreventive potential

Since effective antioxidants can neutralize ROS, therefore they have the inherent capacity to prevent or retard the DNA altering effects of genotoxic agents. In order to test this possibility, the chemopreventive effect of SY was evaluated in a two-stage skin carcinogenesis model using BALB/c mice (albino, lab-bred strain of house mouse). Mice were exposed to 40 nmol of DMBA followed by 4 nmol of TPA twice a week for a duration of 21 weeks.

**Fig. 6:**
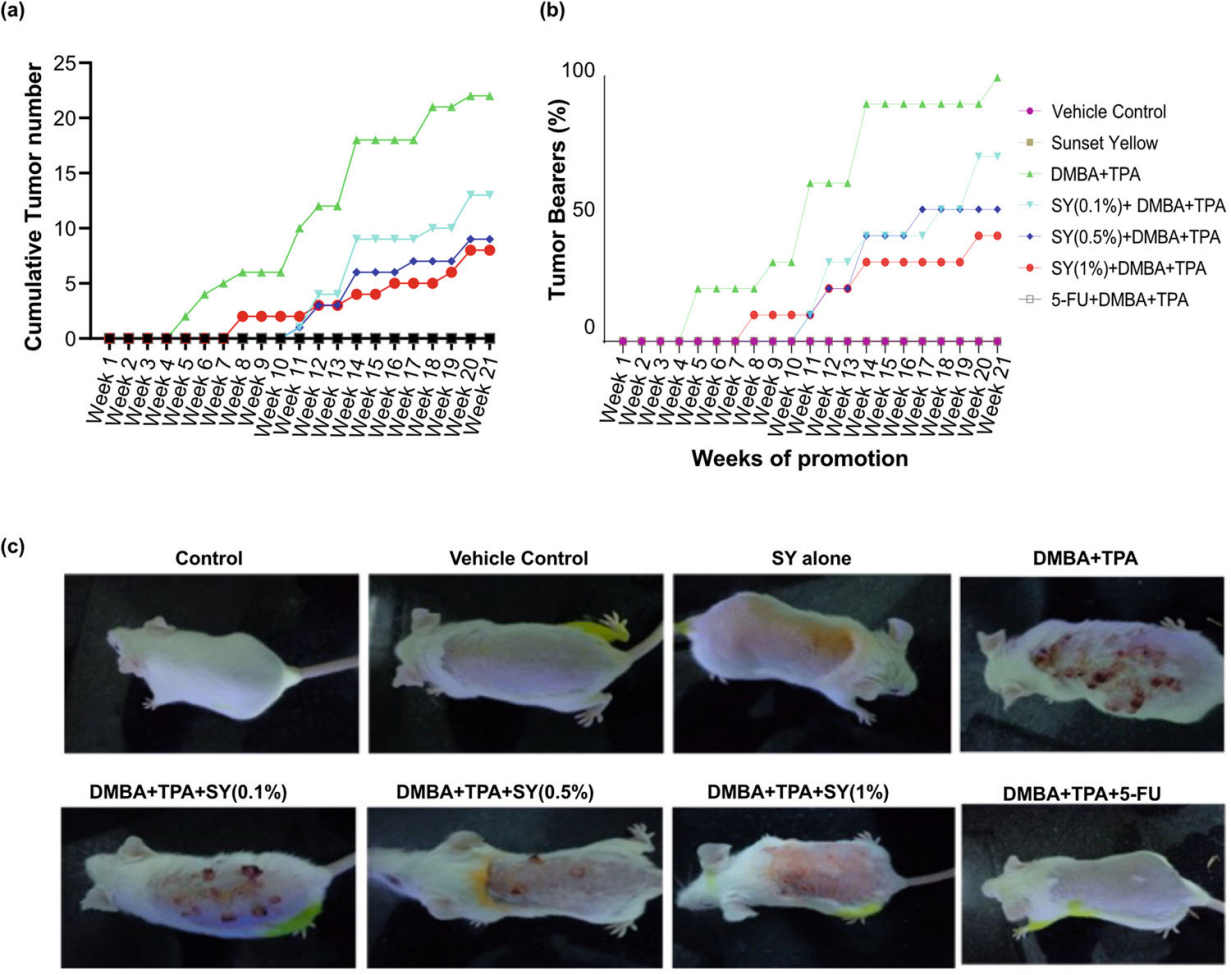
Protective effect of sunset yellow (SY) in a two-stage mouse skin carcinogenic model induced by DMBA and TPA. **a** Cumulative Number of Tumors per Group (*n* = 10) at the end of the 21st week promotion period. **b** Percentage of Mice with Tumors at the end of the 21st week promotion period. **c** Representative photographs were captured at end of 21st week to illustrate the inhibitory effect of SY on DMBA/TPA-induced skin tumorigenesis in BALB/c mice at the conclusion of the treatment.

SY treatment significantly reduced tumor incidence and tumor multiplicity. The latency period was extended by three weeks in the SY-treated groups compared to the DMBA/TPA group. The cumulative number of tumors decreased dose-dependent: from 22 in the DMBA/TPA group to 13, 11, and 8 in the SY-treated groups at doses of 0.1%, 0.5%, and 1%, respectively (Fig. 6a). By the end of the study, all mice in the DMBA/TPA group had developed tumors, whereas the proportion of mice with tumors decreased in the SY-treated groups: 70%, 50%, and 40% for the 0.1%, 0.5%, and 1% SY doses, respectively (Fig. 6b). It was noteworthy that the number of mice with no tumors increased with increasing doses of SY by the end of the study period: 3, 4, and 6 in 0.1%, 0.5%, and 1% SY treated groups, respectively (Table 2). The mean tumor number per mouse was significantly lower in the SY-treated groups compared to the DMBA/TPA group: 2.2, 1.3, 1.1, and 0.8 tumors per mouse for the DMBA/TPA group, 0.1%, 0.5%, and 1% SY group, respectively (Table 3). 5-Fluorouracil (5-FU), an anti-skin cancer drug was used as benchmark molecule for anti-tumor effects. As expected, the animals treated with 5-FU and vehicle control groups of mice did not exhibit any signs of tumor formation.

**Table 2:** Number of mice without tumor after 21-weeks from promotion in different groups

Groups	Number of mice
Vehicle Control	10
Sunset Yellow	10
DMBA + TPA	0
SY (0.1%) + DMBA + TPA	3
SY (0.5%) + DMBA + TPA	4
SY (1%) + DMBA + TPA	6
5-FU + DMBA + TPA	10

**Table 3:** Average number of tumors per mouse from week 5 to week 21

Week of Promotion	5	8	11	14	17	20	21
Vehicle Control	0	0	0	0	0	0	0
Sunset Yellow	0	0	0	0	0	0	0
DMBA + TPA	0.2 ± 0.13	0.5 ± 0.40	1 ± 0.39	1.8 ± 0.44	1.8 ± 0.44	2.2 ± 0.53	2.2 ± 0.49
SY (0.1%) + DMBA + TPA	0	0	0.1 ± 0.10**	0.9 ± 0.43*	1 ± 0.42	1.3 ± 0.47	1.3 ± 0.47
SY (0.5%) + DMBA + TPA	0	0	0.1 ± 0.10**	0.5 ± 0.30**	0.5 ± 0.30**	1 ± 0.44*	1.1 ± 0.43*
SY (1%) + DMBA + TPA	0	0.2 ± 0.20	0.3 ± 0.21*	0.4 ± 0.22**	0.5 ± 0.30**	0.8 ± 0.38*	0.8 ± 0.38**
5-FU + DMBA + TPA	0	0	0	0	0	0	0

Values are mean ± SEM, *n* = 10, Statistical significance isindicated as *P < 0.05, **P < 0.01, and ***P < 0.001 vs. DMBA+TPA-treated group.

These results demonstrate the strong chemopreventive potential of SY, as indicated by the reduction in tumor incidence, tumor multiplicity, and delayed tumor onset. The dose-dependent effects further support SY as a promising agent for cancer chemoprevention.

### In silico profiling of SY targets suggests its mode of action as an antioxidant and chemopreventive agent

We performed computational toxicity analysis (Ames Mutagenicity protocol) of SY using the ‘TOPKAT’ protocol available in the Biovia Discovery Studio v.2022 (DS2022). Our results show that the calculated probability of mutagenicity of SY is zero, suggesting that SY does not have mutagenic properties, confirming the experimental outcomes.

**Fig. 7:**
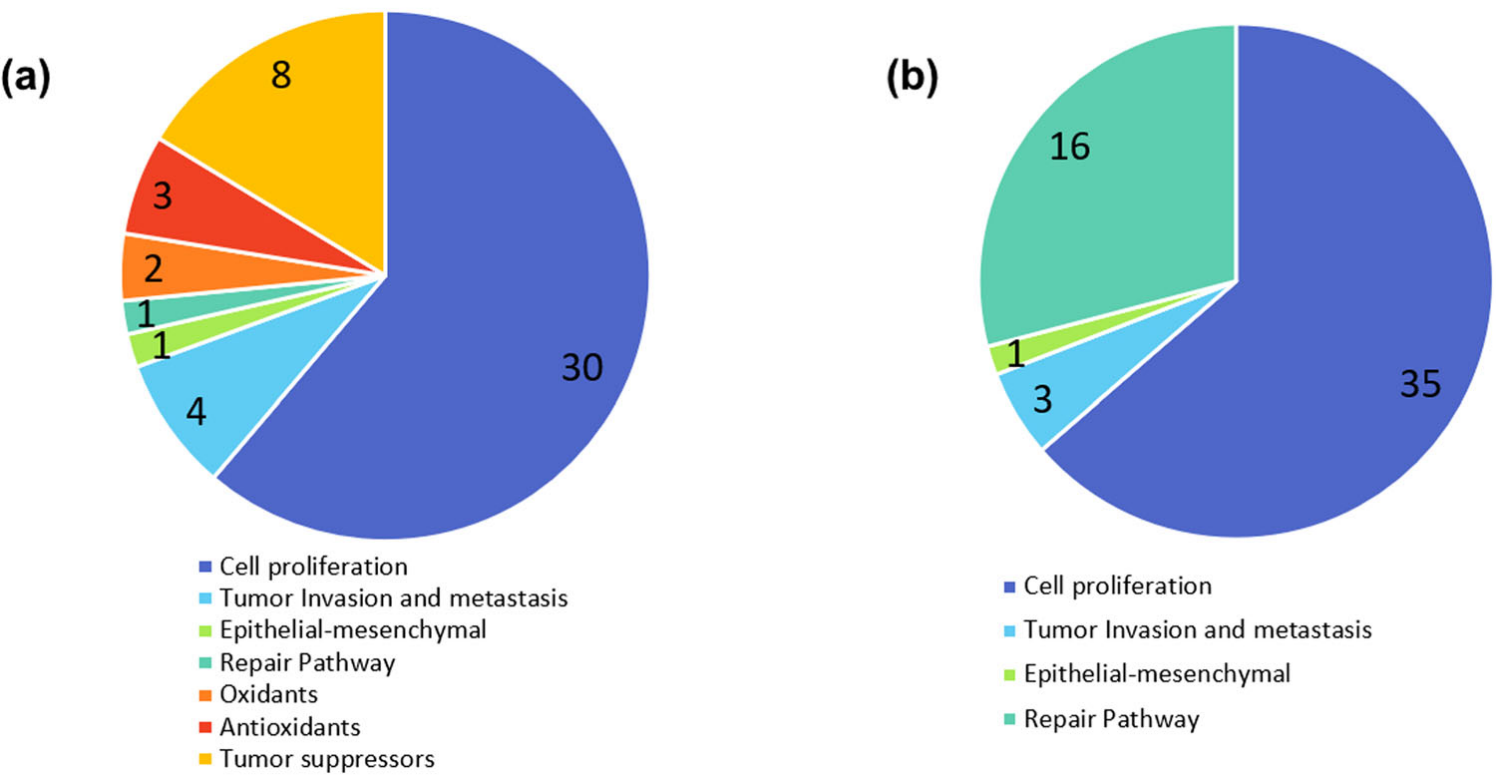
Venn diagrams of potential SY target proteins with a FitValue more than 0.3 in the pharmacophore screening. **a** Target proteins associated with skin cancer; and (**b**) Target proteins associated with other cancers. Detailed information on these target proteins is provided in Supplementary Table 2.

In silico profiling for SY biological targets was carried out using the ‘Ligand Profiler’ protocol available in DS2022, which map the molecule to a set of pharmacophores present in the PharmaDB. The PharmaDB database contains over 250,000 pharmacophores models derived from 16,304 entries from the 2017 release of sc-PDB protein data bank (http://bioinfo-pharma.u-strasbg.fr/scPDB). In total, 4982 pharmacophore models were screened for SY out of over 250,000 pharmacophore models available in PharmaDB database using the ‘Ligand Profiler’ protocol available in the DS2022. Among them, 629 pharmacophore models are from 238 unique proteins from human/murine models (Supplementary Table 1). The top ten potential SY targets based on the decreasing order of Fit value are shown in Table 4. As SY is shown to possess good chemopreventive properties in two-stage mouse skin carcinogenic model, we investigated the role of SY potential target proteins in skin cancer and other tumor-related processes and pathways (Fig. 7).

**Table 4:** Top 10 unique biological targets of SY identified the screening of the PharmaDB database

Rank	PDB ID	Target Name	Target Full Name	Target Class	Fit Value
1	2PVY	FGFR2	Fibroblast growth factor receptor 2	Tyrosine-protein kinase	0.978146
2	3NI5	CAH2	Carbonic anhydrase 2	Lyase	0.970602
3	2ITX	EGFR	Epidermal growth factor receptor	Tyrosine-protein kinase	0.969743
4	3×03	PI42B	Phosphatidylinositol 5-phosphate 4-kinase type-2 beta	Transferase	0.957436
5	4GYZ	TYDP2	Tyrosyl-DNA phosphodiesterase 2	Nuclease	0.9252
6	2EVA	M3K7	Protein-tyrosine kinase 2-beta	Tyrosine-protein kinase	0.923951
7	2G2F	ABL1	Tyrosine-protein kinase ABL1	Tyrosine-protein kinase	0.923912
8	2YP0	CN37	2’,3’-cyclic-nucleotide 3’-phosphodiesterase	RNA-binding	0.919039
9	4JWL	CYTH2	Cytohesin-2	Guanine-nucleotide releasing factor	0.914928
10	4NIE	RORG	Nuclear receptor ROR-gamma	DNA-binding	0.906921

**Fig. 8:**
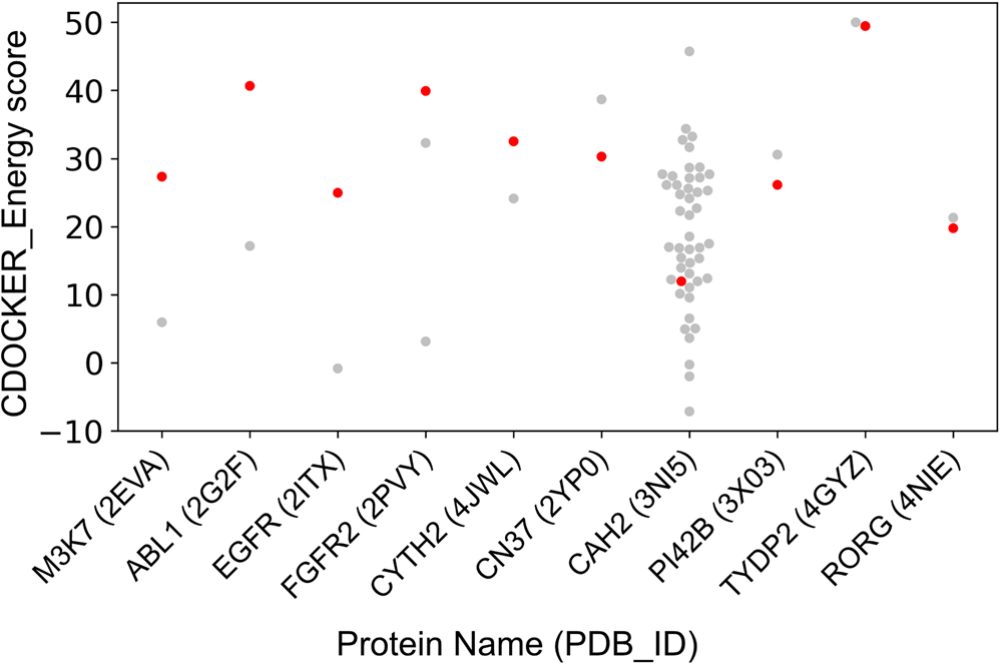
Comparison of the binding affinity of SY with experimentally validated ligands on selected target proteins. The binding affinity is calculated as the CDOCKER_Energy score (negative of CDOCKER energy), where higher value indicates more binding affinity. The gray dots correspond to the original ligands in the PDB file, while the red dots correspond to SY CDOCKER_Energy score with the corresponding target proteins.

For each biological target highlighted in Table 4 and additional target proteins potentially involved in oxidative stress and DNA repair pathways, we compared the binding affinity of SY with experimentally validated ligands bound to the target proteins in the scPDB database. For this, we used CDOCKER, which is an implementation of a CHARMm based docking method^24^ in DS2022. The CDOCKER interaction energy is derived from the CHARMm forcefield energy function, the nonbond potential (vdW and electrostatic) between the ligand and the protein. We used CDOCKER_Energy score, which is the negative of CDOCKER_Energy value, to show the binding affinity between the protein and ligand. The higher the CDOCKER_Energy score, the greater the binding affinity between the protein and ligand. As shown in Fig. 8, SY has a higher CDOCKER_Energy score than the experimentally bound ligands on five out of the top ten target proteins: M3K7 (PDB ID: 2EVA), ABL1 (2G2F), EGFR (2ITX), FGFR2 (2PVY), and CYH2 (4JWL). Therefore, it could be inferred that SY could have a higher regulatory impact on the target proteins compared to the experimentally bound ligands. The top target identified for SY is FGFR2 (fibroblast growth factor receptor 2), which is essential in the regulation of cell proliferation, differentiation, migration, and apoptosis. Aberrant FGFRs signaling has been implicated in various cancer types, which motivated the development of selective FGFR2 inhibitors^25,26^. Our results indicate that SY has a much higher binding affinity with two experimentally validated FGFR2 inhibitors, ACP^27^ and M33^28^.

Similarly, EGFR (epidermal growth factor receptor) inhibitors are very well explored for the treatment of several cancers, including colorectal, head and neck, lung, and pancreatic cancer. In our docking analysis, SY showed better binding affinity in comparison to experimentally validated ligands of EGFR (PDB ID: 2ITX). Our results indicate that the protective effect of SY in mouse skin carcinogenic models induced by DMBA and TPA may be through the inhibition of FGFR2 and EGFR.

The carbonic anhydrase 2 (CAH2) inhibitors have previously been shown to reverse the ROS in mouse cerebral pericytes and possess antioxidant properties^29–31^. We compared the binding affinity of SY along with 46 previously known CAH2 inhibitors and found that SY has better affinity than at least 10 previously known inhibitors. These results suggest that the antioxidant property of SY might be due to the inhibition of CAH2.

The NRF2-ARE (nuclear factor *erythroid 2-related factor 2) pathway is very well investigated as an intrinsic mechanism of defense against oxidative stress. Considering its potential role against tBHP-induced DNA damage (Fig. 4) and DPPH radical scavenging activity (Fig. 5), we further explored potential target proteins of SY in the NRF2-ARE pathway. Interestingly, we found two proteins, Glycogen synthase kinase-3 beta (GSK3β) and Kelch-like ECH-associated protein 1 (KEAP1), as potential targets of SY with FitValue 0.77 and 0.53. Inhibitors of KEAP1 are known to disrupt the covalent interactions between KEAP1 and NRF2 to initiate the cellular antioxidant and detoxification process^32^. Similarly, GSK3β has also been established as a potential therapeutic target that modulates NRF2 signaling cascade^33^. Comparing the binding affinity of SY with both KEAP1 and GSK3β, we found that SY has better efficacy with many of the previously known inhibitors. These results suggest that the protection against DNA damage and the antioxidant properties of SY might be due to the inhibition of CAH2, KEAP1, and GSK3β. While a high FitValue can indicate a good binding affinity, the ratio of pharmacophore models of a certain protein being flagged as a potential target by screening over the total number of pharmacophores available for that protein in the scPDB database can be a better indication of the top targets. Indeed, some proteins have a large pool of available experimental structures in the database as they are largely studied (for example, human CDK2 (Cyclin Dependent Kinase 2) protein has 274 entries in the scPDB database). In contrast, others only have one or two database entries. Therefore, we could postulate that 5 structures flagged as targets out of 100 available structures for a particular protein might be less reliable than 5 potential targets out of 10 available structures for a different protein. This ratio was normalized in order to be comparable between proteins with different amounts of scPDB entries as shown in Eq. 2 below:

2 $${{AR}}_{P}=\frac{\frac{{s}_{P}}{{T}_{P}}-\frac{1}{{T}_{P}}}{1-\frac{1}{{T}_{P}}}\,{\mathrm{for}}\,{\mathrm{T}}_{\mathrm{P}} > 1{{;}}\,{{AR}}_{P}=0.5\,\text{for}\,{T}_{P}=1$$

Where ${{AR}}_{P}$ is the adjusted ratio of a given protein $P$; ${s}_{{P}}$ is the number of entries screened as targets in pharmacophore screening process; ${T}_{P}$ is the total number of entries in the scPDB database for the protein $P$.

An “adjusted ratio” of 0 indicates that just one entry for that protein was examined as a potential target from among all existing entries; a value of 1 means that all existing entries for this UniProt ID were flagged as potential targets during screening. For proteins, with only one available entry in the scPDB database, the adjusted ratio value was set to 0.5.

**Fig. 9:**
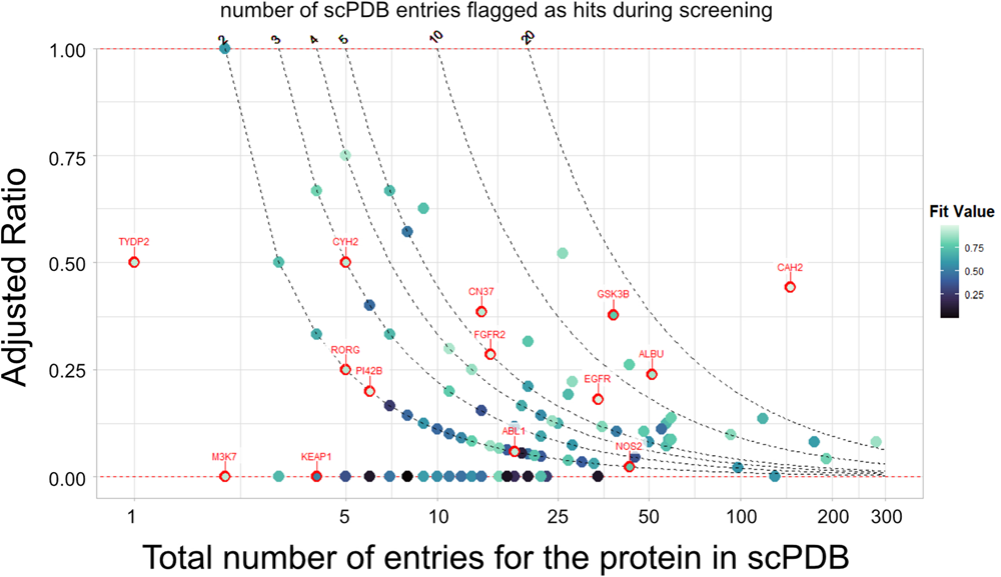
Plot of the adjusted ratio of protein entries screened as potential targets over to the total number of entries in the scPDB database. The red dotted lines represent the lowest and highest possible adjusted ratio values (0 and 1). The top target proteins associated with SY antioxidant and chemopreventive properties are labeled. The dots are colored based on the FitValue score of the protein.

As shown in Fig. 9, the protein among the 14 proteins of interest which comparatively has the highest number of screening hits is human CAH2 protein, which also has the highest number of entries in the scPDB database. It is important to note that this adjusted ratio is not well-suited for proteins with very low database entries count, like mouse TYDP2 (1 entry) or human M3K7 (2 entries). These results suggest that CAH2 protein binding and inhibition by the SY might be the best hypothesis to explain its antioxidant properties.

### Experimental validations of computationally predicted SY targets

**Fig. 10:**
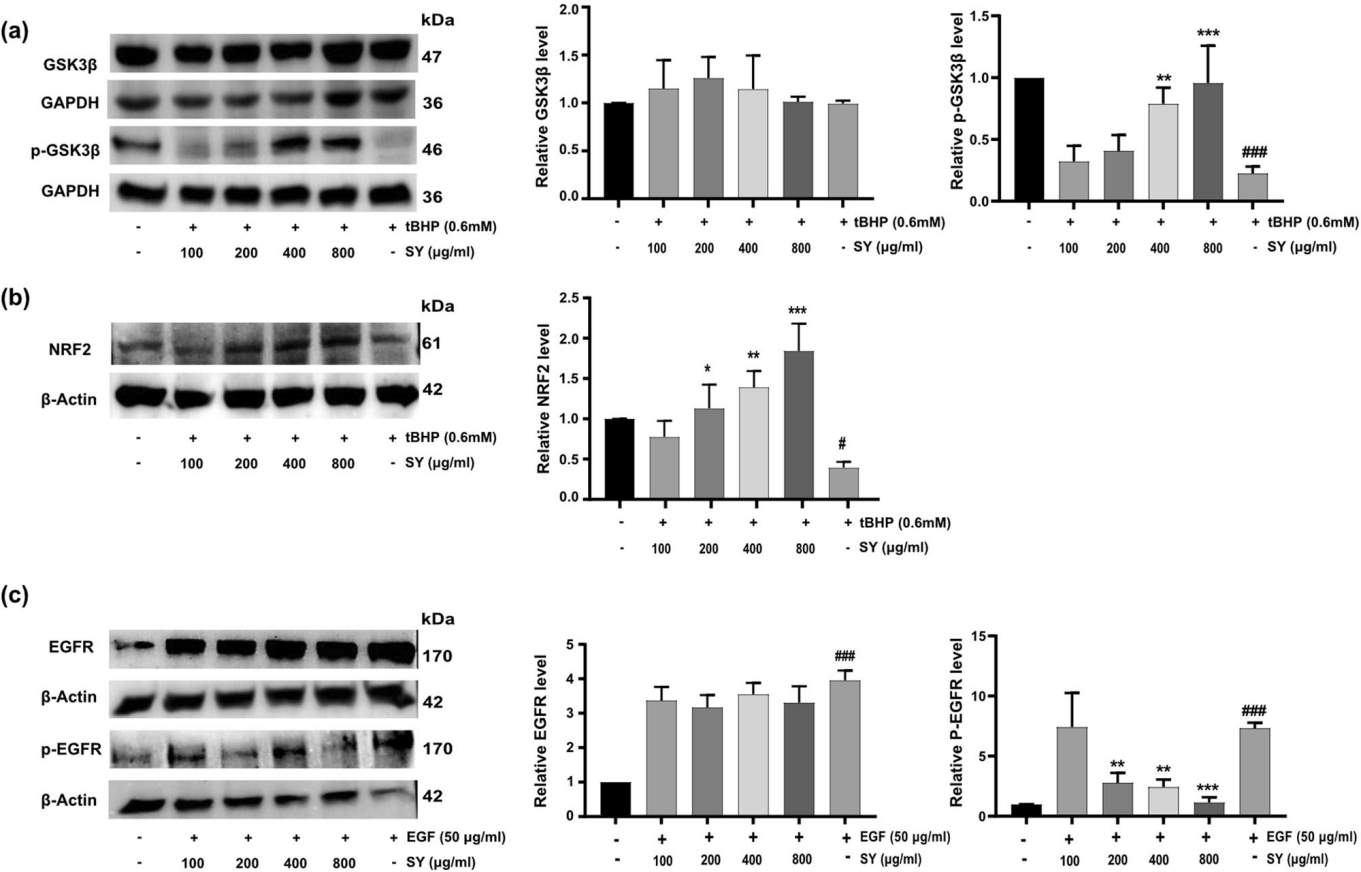
Effect of pretreatment of SY on p-GSK3β, NRF2 and p-EGFR on tBHP and EGF stimulated HaCaT cells. HaCaT cells were treated with various concentrations of SY for 12 h, followed by exposure to 0.6 mM tBHP for 6 hours. The total cell lysates were prepared, and the protein expression of p-GSK3β (**a**) and NRF2 (**b**) was examined by Western blot. GAPDH and β-Actin were used as loading controls for extracts, respectively. Scanning densitometry was used for analysis compared with control groups (*n* = 3). For analysis of inhibition of EGFR phosphorylation, HaCaT cells were pretreated with SY (100–800 μg/ml) for 12 h followed by 30 min exposure to EGF (50 ng/ml). The cells were then lysed and prepared for western blotting using antibodies against EGFR, phosphor EGFR (p-EGFR), and β actin (**c**). The protein levels were assessed by western blotting and quantified by densitometric analysis (*n* = 3). The differences among the groups were analyzed with one-way ANOVA followed by the Tukey post hoc test. Results are presented as Mean ± standard error, combining data from three independent experiments (*n* = 3). Statistical significance: ^#^*P* < 0.05, ^###^*P* < 0.001 vs. untreated control; *P < 0.05, **P < 0.01, and ***P < 0.001 vs. tBHP-treated or EGF-treated cells.

Next, the putative molecular targets of SY identified by in silico study were validated through immunoblotting. Glycogen synthase kinase-3β (GSK3β) acts as a negative regulator of NRF2 by encouraging NRF2 degradation. The phosphorylation of GSK3β has facilitates the nuclear accumulation of NRF2 and the expression of genes that counteract oxidative stress. The HaCaT cells were pretreated with SY for 12 h, followed by exposure to tert-butyl hydroperoxide (tBHP) for an additional 6 h. The immunoblotting data clearly demonstrated a dose-dependent enhancement of phosphorylated GSK3β in SY treated groups (Fig. 10a).

KEAP1, an actin-binding protein, also regulates NRF2 by selectively binding to its amino-terminal regulatory domain which retains NRF2 in the cytoplasm. In this way, KEAP1 acts upstream to NRF2, and inhibition of KEAP1 would downregulate the expression of NRF2. The separation of NRF2 from KEAP1 is facilitated by antioxidants which enables nuclear accumulation of NRF2 and upregulation of cytoprotective genes. Immunoblot analysis of NRF2 expression revealed a marked decrease in NRF2 protein levels in the tBHP-treated group compared to the untreated control group. However, SY treatment significantly upregulated NRF2 expression in a dose-dependent manner. These findings suggest that SY can effectively counteract tBHP-induced NRF2 suppression and promote NRF2 activation, thereby enhancing cellular antioxidant defense mechanisms. (Fig. 10b).

In another set, EGF stimulated HaCaT cells with high expression of p-EGFR were used to study the effect of SY on p-EGFR expression. It is established that aberrant cancer cell proliferation is associated with EGFR overexpression. EGF treatment induced a significant increase in p-EGFR expression. However, pretreatment with SY notably suppressed EGF-induced p-EGFR expression in HaCaT cells. These findings suggest that SY can effectively inhibit EGFR activation, a key step in cancer cell proliferation (Fig. 10c).

## Discussion

In the current study, we aimed to investigate the protective and antioxidant properties of Sunset Yellow (SY) in human keratinocyte-derived HaCaT cells and its potential as a chemopreventive agent in a mouse model of skin carcinogenesis induced by DMBA/TPA. In vitro experiments using HaCaT cells revealed that pretreatment with SY effectively ameliorated the cytotoxic effects induced by tBHP, a well-established cytotoxic agent known to induce oxidative stress. We observed a significant dose-dependent improvement in cell viability and significant protection against tBHP-induced morphological alterations upon SY administration. PCNA, a protein actively expressed in proliferating cells, plays a crucial role in facilitating DNA replication and repair processes, contributing to overall cellular health^34^. Our findings indicate a reduction in PCNA expression in HaCaT cells following tBHP treatment. Notably, cells pretreated with SY and subsequently exposed to tBHP exhibited an enhanced expression of PCNA, indicative of a healthier cellular physiology. Overall, these findings underscore the cytoprotective potential of SY against oxidative damage in HaCaT cells. Furthermore, we investigated the effect of SY on the intracellular generation of reactive oxygen species (ROS) in HaCaT cells exposed to tBHP. Remarkably, tBHP exposure resulted in a substantial increase in intracellular ROS levels, indicative of heightened oxidative stress. However, pretreatment with SY demonstrated a dose-dependent reduction in intracellular ROS generation, highlighting its antioxidant capacity. Free radicals, characterized by one or more unpaired electrons, including ROS, play a detrimental role in various skin pathologies including skin carcinogenesis^35^. Excessive ROS production disrupts the redox balance, leading to oxidative stress, DNA damage, gene mutations, and increased cell proliferation, ultimately contributing to skin carcinogenesis^9,36^. The ability of SY to scavenge ROS suggests its potential to restore redox balance and confer protection against oxidative damage. Mitochondrial dysfunction, often associated with excessive ROS generation, can contribute to cellular damage. Thus, we evaluated the mitochondrial membrane potential (MMP) in HaCaT cells under different treatment conditions. Our findings revealed that tBHP treatment led to a significant decline in MMP, signifying mitochondrial impairment. However, pretreatment with SY mitigated the tBHP-induced decline in MMP, suggesting its role in preserving mitochondrial function and integrity. Antioxidants are known to inhibit ROS-dependent cellular toxicity, weaken chain initiation reactions, and impair free radical activity^37^. In our study, we observed that SY demonstrated antioxidant activity by protecting HaCaT cells from cytotoxicity, excessive ROS production, and mitochondrial dysfunction induced by tBHP. This is of particular significance considering that an imbalance in ROS formation leads to oxidative stress, which is implicated in oxidative DNA damage, skin pathologies and skin cancer development^35,36^.

To assess the protective effects of SY against genotoxic damage, we examined its impact on tBHP-induced DNA damage in HaCaT cells. Our results demonstrated that SY pretreatment effectively reduced DNA damage, as evidenced by decreased tail DNA length and olive tail moment (OTM) in the alkaline comet assay. These findings imply that SY has the potential to safeguard DNA against oxidative damage induced by tBHP. Additionally, we observed a significant decrease in micronucleus formation, a marker of chromosomal damage and genotoxicity, in the SY-treated groups compared to the tBHP group.

DNA double-strand breaks, if left unrepaired, can lead to genomic instability and potentially contribute to cancer development. The phosphorylation of γ-H2AX is essential for cells to recognize and mount a response to DNA damage. In our study, tBHP-treated HaCaT cells demonstrated an elevated expression of γ-H2AX, indicative of significant DNA damage. However, cells treated with a combination of tBHP and SY exhibited a dose-dependent reduction in the expression of γ-H2AX. This observed decrease in γ-H2AX expression suggests that SY may mitigate tBHP-induced DNA damage. Overall, these findings emphasize the potential protective effect of SY in attenuating DNA damage and highlights its role in maintaining genomic stability under conditions of cellular stress induced by tBHP.

Furthermore, the antioxidant activity of SY was assessed by evaluating its standard reduction potential (${E}^{o}$) and DPPH scavenging activity. Notably, we found that SY exhibited E° and significant DPPH scavenging activity, comparable to that of ascorbic acid, a well-known antioxidant. Our results show that SY possesses a strong standard reduction potential of 0.211 V, and interestingly most of the principal antioxidants present in our food stuffs have a reduction potential in the range of 0.2–0.6 V^38^. Overall, these findings indicate the potent antioxidant properties of SY and its potential to mitigate oxidative stress-related conditions. Previous studies have highlighted the significant role of ROS in skin carcinogenesis, as they induce DNA damage and gene mutations. ROS primarily targets the electron-rich bases of DNA, causing oxidation and making them susceptible to genetic modifications^39^. In the in vivo component of our study, we evaluated the chemopreventive effect of SY using a mouse model of DMBA/TPA-induced skin carcinogenesis. The DMBA/TPA model represents a well-established experimental approach for studying skin cancer development. DMBA acts as an initiator, while TPA serves as a promoter, mirroring the natural progression of human tumor development through initiation, promotion, and progression phases. This model has been extensively utilized to investigate the chemopreventive potential of drugs and explore molecular pathways involved in tumor progression. Notably, the similarity of gene alterations between mouse and human skin cancer indicates a biological analogy, further supporting the relevance of this model for studying the impact of chemopreventive agents on human cancer development^40^. Administration of SY resulted in a significant reduction in tumor incidence and tumor multiplicity compared to the DMBA/TPA group. Notably, we observed an extended latency period for tumor development in the SY-treated groups, indicating a delay in tumor onset. Furthermore, the cumulative number of tumors decreased in a dose-dependent manner with increasing concentrations of SY. Chemotherapy and radiotherapy are currently the primary treatments for cancer; however, they are often associated with side effects and challenges in achieving optimal outcomes^41^. Therefore, there is a pressing need for chemopreventive agents that possess minimal long-term toxicity, effectively reduce tumor onset, delay latency, or inhibit tumor promotion. Clearly, our study demonstrated chemopreventive efficacy of SY in reducing tumor incidence and burden in a DMBA/TPA-induced skin carcinogenesis model.

To understand the mode of action of SY as antioxidant, anti-genotoxic, and cytoprotective agents, we screened potential biological targets using computational pharmacophore screening methods. Structure-based pharmacophore virtual screening methods are successfully used in the past for target identification, off-target prediction, toxicity prediction, drug repositioning^42–48^. Through pharmacophore screening, several target proteins are identified, including fibroblast growth factor receptor 2 (FGFR2), epidermal growth factor receptor (EGFR), carbonic anhydrase 2 (CAH2), glycogen synthase kinase-3 beta (GSK3β), and Kelch-like ECH-associated protein 1 (KEAP1). The study compares the binding affinity of SY with experimentally validated ligands for these proteins and suggests that SY may have a higher regulatory impact on the target proteins compared to the known ligands. This analysis indicates that SY may inhibit FGFR2, EGFR, CAH2, KEAP1, and GSK3β, which are involved in various cellular processes related to cancer development and oxidative stress response.

Nuclear factor erythroid 2-related factor 2 (NRF2) is a transcription factor that modulates the expression of genes involved in oxidative stress response. NRF2 modulates both basal and induced expression of a spectrum of genes dependent on antioxidant response elements, thereby influencing the physiological and pathophysiological consequences of exposure to oxidants. Additionally, NRF2 contributes to the mitigation of inflammatory damage by regulating inflammatory factors^49,50^. Both Glycogen synthase kinase 3β (GSK3β) and Kelch-like ECH-associated protein 1 (KEAP1) serve as negative regulators of the cellular antioxidant response, with NRF2 acting as the central orchestrator of this regulatory mechanism. GSK3β enforces a negative regulatory influence on NRF2 by governing its subcellular localization. GSK3β undergoes inhibition through phosphorylation at Ser9 by Ser/Thr protein kinases, resulting in up-regulation of NRF2^51,52^. Similarly, under normal conditions, KEAP1 binds to NRF2 in the cytoplasm, preventing NRF2 translocation to the nucleus^53^. However, upon exposure to oxidative stress, oxidants molecules modify cysteine residues on KEAP1, which induces conformational changes thereby deactivating KEAP1-dependent negative regulation of NRF2. The functional inactivation of KEAP1 stabilizes NRF2 and triggers the expression of cytoprotective genes^53,54^. Since, our in silico study predicted GSK3β and KEAP1 as important target of SY, therefore, we investigated the effect of SY pretreatment on GSK3β and NRF2 expression in tBHP-treated HaCaT cells. We found that SY induced GSK3β phosphorylation at Ser9 and enhanced expression of NRF2 in tBHP-treated HaCaT cells. These findings suggest that enhanced NRF2 upregulation might be a consequence of GSK3β phosphorylation and inhibition of KEAP1 by SY.

EGFR was yet another putative in silico target of SY observed in our study. EGFR is well known to be overexpressed concurrently with abnormal cancer cell proliferation^55^. Growth factor binding to EGFR results in EGFR phosphorylation, which activates an adapter molecule complex that causes the transcription of many genes to be induced, which are essential for cell survival and proliferation^56^. Notably, pretreatment with SY dramatically downregulated the EGF-induced expression of p-EGFR, in comparison to cells treated with EGF alone. The present study provides compelling evidence that SY enhances cellular antioxidant defenses and exhibits chemopreventive properties. The observed effects of SY may be attributed to its ability to interact with key molecular targets, including GSK3β, KEAP1, and EGFR. These interactions may contribute to the modulation of signaling pathways involved in oxidative stress and cancer development, suggesting SY’s beneficial potential for skin cancer and other inflammatory skin pathologies.

Overall, the study demonstrates the significant antioxidant, anti-genotoxic and cytoprotective activity of SY with an ability to scavenge free radicals. Moreover, the findings from the two-stage skin carcinogenesis model suggest that SY has potential as a chemopreventive agent by reducing tumor incidence, multiplicity, and onset. The computational analysis and their in vitro validations provide insights into the potential mode of action of SY, suggesting its interaction with key proteins involved in cancer and oxidative stress-related pathways. These findings contribute to our understanding of SY’s potential applications in combating oxidative stress-related conditions and its role as a chemopreventive agent.

## Methods

### Chemicals and reagents

The Sunset Yellow FCF was purchased from Hickson & Dadajee Private Limited in Mumbai. Fetal bovine serum (FBS) was purchased from Gibco, qualified, Brazil. Penicillin/streptomycin (Cat. No: 15240062: Thermo Fisher), Propidium Iodide (PI), 2′,7′ -dichlorodihydrofluorescein diacetate (DCFH_2_-DA) were purchased from Invitrogen (Carlsbad, CA, USA), JC-1 dye was procured from ENZO, Germany. Trypsin-EDTA solution (0.25%), phosphate buffers, and tween-20 were bought from Sigma Aldrich company (St. Louis, MO, USA). 7,12-dimethylbenz [a]-anthracene and *12-O-Tetradecanoylphorbol-13-acetate* (TPA) were purchased by Sigma-Aldrich. The other chemicals (PCNA (10004805), λH2AX (D7T2V), EGFR (#2232 S), PEGFR (#2235 S), NRF2 (sc13032), GSK3β (#610202), and PGSKβ (sc-11757)) were all the highest purity grades and were purchased from Sigma-Aldrich unless otherwise stated.

### Cell culture

The HaCaT cell line, derived from human skin keratinocytes, was procured from the AddexBio located in San Diego, USA The cells were subcultured and maintained in Dulbecco’s Modified Eagle Medium (DMEM) obtained from Sigma-Aldrich (Cat. No. D-8900). The culture medium consisted of 10% FBS and 1% penicillin-streptomycin. The cells were cultured in a CO_2_ incubator (37 °C temperature, 95% relative humidity and 5% CO_2_ atmosphere). The experiments were initiated when the cell cultures reached approximately 70% confluency, ensuring an optimal cellular density for conducting the study.

### Cell viability assay

Cell viability was assessed using propidium iodide (PI) staining-based cytotoxicity assay in HaCaT cells. The cells were seeded at a density of 2 × 10^5^ cells/well in 6-well plates. After a 24-hour incubation period, the cells were treated with varying concentrations of SY (100–800 μg/ml) for an additional 24 h. In another set, to investigate the impact of pre-treatment with SY on cell viability, the cells were pre-treated with SY (100–800 μg/ml) for 12 h prior to exposure to 0.6 mM tert-butyl hydroperoxide (tBHP) for 6 h. Before analysis, the cells were washed twice and subjected to PI staining. The stained cells were then analyzed using flow cytometry (FACS Canto II, BD Biosciences, San Jose, CA) within 30 min of staining.

### Cell morphology evaluation

HaCaT cells were cultured in DMEM media at a seeding density of 2 × 10^5^ cells per well. The cells were then subjected to pretreatment with various doses of SY (100–800 µg/ml) for 12 h. Following the pretreatment, the cells were exposed to tBHP for 6 h at a temperature of 37 °C in a 5% CO_2_ atmosphere. Subsequently, the cells were observed and analyzed using an EVOS M5000 microscope from Thermo Fisher Scientific.

### Measurement of intracellular ROS generation and mitochondria membrane potential (MMP)

Dichloro-dihydro-fluorescein diacetate (DCFH_2_-DA) was used to monitor ROS generation in HaCaT cells. DCFH_2_-DA is a dye used for the relative quantification of ROS generation in cells. The cellular esterase activity breaks the DA moiety, which makes the cell membrane impervious to DCF dye. In the presence of ROS, DCF interacts with reactive oxygen species (ROS) and emits green fluorescence^57^. We seeded HaCaT cells at a density of 2 × 10^5^ cells per well. For a period of 12 h prior to tBHP treatment, the cells were pretreated with SY at doses ranging from 100–800 µg/ml. Subsequently, the cells were exposed to 0.6 mM tBHP for 6 h. After the treatment, the cells were incubated with 10 µM DCFH_2_-DA for 30 min at a temperature of 37 °C. Following the incubation, the cells were washed three times with PBS and resuspended in PBS. The fluorescence intensity of DCF was measured using flow cytometry (for gating strategy see Supplementary Fig. 1).

In order to detect the mitochondrial depolarization in HaCaT cells, JC-1 dye was used. JC-1 dye shows a red fluorescence in healthy cells with high MMP, whereas it exhibits a green fluorescence in unhealthy cells (cells with membrane depolarization) with low MMP. In another set, the cells were pretreated with SY at concentrations ranging from 100 to 800 μg/ml for a period of 12 h before being exposed to tBHP. Subsequently, the cells were subjected to 0.6 mM tBHP for another 6 h. Next, the trypsinized cells were pelleted down, and the supernatant was discarded. Then after, the cells were resuspended in PBS, followed by the addition of JC-1 dye (5 µM) for staining the cells, and incubated for 15 min. The cells were analyzed by BD FACS flow cytometry (for gating strategy see Supplementary Fig. 2).

### The alkaline single-cell gel electrophoresis assay (Comet assay)

The alkaline single-cell gel electrophoresis (SCGE) assay, commonly known as the comet assay, was employed to evaluate DNA strand breaks following a methodology adapted from Dubey et al.^58^. HaCaT cells were cultured in a 6-well plate at a density of 2 × 10^5^ cells/ml. Cells were pre-treated with SY for 12 h, followed by treatment with tBHP for 6 h. Subsequently, the cells were harvested and suspended in 150 µL of phosphate-buffered saline (PBS). Frosted-end glass slides (75 mm × 25 mm) were coated with 1% NMA and air-dried at room temperature for 24 h. A cell suspension of 100 µL was mixed with 100 µL of 1% low melting point agarose (LMPA). An 80 µL aliquot of this mixture was carefully layered onto two pre-coated glass slides, covered with a coverslip, and placed on ice for 10 min to allow the agarose to solidify. Subsequently, an additional layer of 90 µL of 0.5% LMPA was applied on top of each slide, covered with a coverslip, and the slides were immersed in a chilled lysing solution. The slides were then incubated at 4 °C overnight for optimal lysis. Following the lysis step, the slides were placed in a horizontal gel electrophoresis tank filled with electrophoresis buffer. The alkaline buffer was used to facilitate DNA unwinding and was allowed to act on the slides for 20 min. Subsequently, electrophoresis was performed at 24 volts for 30 min, with the current adjusted to 300 mA by modifying the buffer volume.

Following electrophoresis, all slides were neutralized and stained with ethidium bromide (EtBr). A fluorescence microscope equipped with Komet software (Komet 5.5, kinetic imaging, U.K.) was utilized to examine and score a total of 50 cells per exposure (25 cells per slide) at a magnification of 400X. To prevent overexposing comet heads relative to comet tails, exposure duration was modified; comet heads were oriented to the left and comet tails to the right side of the image. The parameters Tail DNA (TD) and the olive tail moment (OTM) were selected for analysis of genotoxicity as reported by Langie et al.^59^ and Simon et al.^59,60^. TD, which is computed as (tail intensity)/ (comet intensity), is a proportional measurement of the quantity of DNA in the comet tail, with respect to the total amount of DNA. Olive tail moment is a more intricate calculation that accounts for both TD and the variations in optical brightness between the comet head and tail. (Tail Mean Intensity-Head Mean Intensity)/100 was used to determine this value. The experiment was conducted in triplicate to ensure robustness and reproducibility of the results.

### Micronucleus assay

Micronuclei formation occurs as a result of acentric DNA fragmentation and loss of chromosomes during mitosis, representing a marker of genotoxicity. In this study, HaCaT cells were seeded at a density of 1 × 10^5^ cells per well in a 6-well plate. Cells were pre-treated with various concentrations of SY (100–800 µg/ml) for 12 h, followed by treatment with tBHP (0.3 mM) for 6 h. Upon completion of the treatment, the treatment medium was removed, and the cells were washed twice with phosphate-buffered saline (PBS). Subsequently, complete media were added to each well, and the cells were allowed to grow for an additional 48 h. After the incubation period, the cells were collected by trypsinization, and the supernatant was discarded. The cell pellet was then resuspended in 1 ml of Solution 1, composed of NaCl (584 mg/L), Igepal (0.3 mL/L), ethidium bromide (25 mg/L), sodium citrate (1000 mg/L), and RNase (10 mg/L). The cell suspension was gently mixed and incubated at room temperature for 1 h. Following the 1-hour incubation, 1 ml of Solution II, containing citric acid (15 mg/L), ethidium bromide (40 mg/L), and sucrose (0.25 M), was added to Solution I. The resulting mixture was then incubated for 15 min. Finally, the cells were analyzed using BD FACS flow cytometry to quantify the presence of micronuclei (for gating strategy see Supplementary Fig. 3). This analysis provides valuable insights into genotoxic effects and chromosome aberrations. The experiment was performed in triplicate to ensure reliable and reproducible results.

### Western blot analysis

Total protein was extracted from HaCaT cells following treatment with varying concentrations of SY (100–800 μg/ml) for 12 h, followed by tBHP (0.6 mM) treatment for 6 h for protein analysis. In another experimental set, HaCaT cells were treated with varying concentrations of SY for 12 h and subsequently treated with EGF (50 ng/ml) for 30 min. The cells were harvested, lysed in RIPA buffer (1% phenylmethylsulfonyl fluoride, PMSF) on ice, and quantified using BCA assay. A total of 50 µg of protein was loaded per lane and separated by SDS-PAGE before transfer onto 0.2-μm polyvinylidene fluoride (PVDF) membranes. Membrane blocking was performed for 1 h at room temperature with 3% BSA. The membrane was probed for 3 h with primary antibodies against PCNA (1:1,000 dilution) (Cat#10004805), were obtained from Cayman Chemical, Ann Arbor, MI. GAPDH (1:1000 dilution) (sc-137179), β-actin (1:1000 dilution) (sc-47778), Phospho-GSK3β (1:1000 dilution) (sc-11757) and NRF2 (1:1000 dilution) (sc13032) from Santa Cruz Biotechnology (Santa Cruz, CA, USA). Antibodies against EGFR (1:1000 dilution) (#2232 S), p-EGFR (1:1000 dilution) (#2235 S), and Gamma-H2AX (D7T2V) (1:1000 dilution) were purchased from Cell Signaling Technology (Beverly, MA, USA), while anti-GSK3β (1:1000 dilution) (#610202) was obtained from BD Biosciences. Subsequently, secondary antibodies, Goat anti-mouse (1:5000) (#31430) and Goat anti-rabbit (1:5000) (#31460), were obtained from Invitrogen, USA, and incubated for 2 h at room temperature. Blot images were acquired and analyzed using the Image Quant LAS 500 imaging system. β-actin (1:1000 dilution) (sc-47778) and GAPDH (1:1000 dilution) (sc-137179) were employed as internal controls. Chemiluminescence detection was executed using a hypersensitive ECL chemiluminescence kit. Image Studio TM lite (LI-COR Biosciences) was utilized for the outcome analysis, and fold increases relative to the control were employed to present the data (The full-length western blot membranes are shown in Supplementary Fig. 4).

### Estimation of standard reduction potential through electrochemical analysis

Electrochemical analysis to measure standard reduction potential (E°) was performed using a three-electrode system. The working electrode consisted of a glassy carbon electrode, while an Ag/AgCl electrode served as the reference electrode, and a platinum wire was used as the counter electrode. This three-electrode setup was immersed in an electrolyte solution containing the solute of interest, either SY dye or ascorbic acid, dissolved in deionized (DI) water with a resistivity of 18 MΩ. The electrolyte solution and electrodes together formed the electrochemical cell. To determine the redox potential of SY dye and ascorbic acid separately, two different sets of experiments were conducted. In each experiment, the solute of interest was introduced into the electrochemical cell. The electrochemical cell was then connected to a potentiostat (Autolab 302 N, Metrohm India Ltd, Netherlands) to measure the standard reduction potential of the solute. Cyclic voltammetry (CV) experiments were performed at a scan rate of 10 mV/s. The CV experiments were conducted at room temperature. During the CV experiment, the potential applied to the working electrode was varied linearly, resulting in a cyclic voltammogram. The cyclic voltammogram displayed oxidation and reduction peaks, which were used to estimate the standard redox potential of the solute. The CV experiments were carried out separately for SY dye and ascorbic acid, and the obtained results were analyzed as discussed ahead in the “result” section. The room temperature was maintained throughout the experiments to ensure consistent and reliable measurements.

### DPPH radical scavenging assay

The DPPH radical scavenging assay was employed to evaluate the free radical scavenging potential of SY as described by Rahman et al.^61^. This assay is based on the ability of the scavenging ability of antioxidants to donate hydrogen atoms, resulting in the fading of the 2,2-diphenyl-1-picrylhydrazyl (DPPH) radical. In brief, a methanolic solution of DPPH at a concentration of 0.5 mM was prepared. Different concentrations of SY (100, 200, 400, and 800 µg/ml) or ascorbic acid (45 µg/ml) were mixed with the DPPH solution. The reaction mixture was thoroughly vortexed and then incubated in darkness for a duration of 30 min to allow the reaction to occur. After the incubation period, the absorbance of the reaction mixture was measured spectrophotometrically at a wavelength of 517 nm.

The DPPH radical scavenging activity was quantified as shown in Eq. 3 below:

3 $${\rm{Scavenging}}\; {\mathrm{activity}}\left( \% \right)=\left(1-\frac{{absorbance}\,{of}\,{sample}}{\,{absorbance}\,{of}\,{control}}\right)* 100$$

This calculation provided a measure of the percentage of DPPH radicals scavenged by SY and allowed for assessing its hydrogen atom donating capability. The experiment was performed in triplicate to ensure the reliability and reproducibility of the results.

### Animal care

BALB/c female mice were used in this study. 6–7 week old female mice (20 ± 3 g) were taken from the animal breeding colony of CSIR-Indian Institute of Toxicology Research (CSIR-IITR), Lucknow. The mice were accommodated under standard laboratory conditions and maintained in the controlled atmosphere of 12-hour dark/light cycle, 22 ^o^C temperature, and 50–60% humidity as per rules approved by the Animal Welfare Committee of CSIR-IITR. All mice were nourished by the food (Alromin Speziallfutler GmbH & Co, Germany) and water *ad libitum*. For all experiment *Permission* were granted by the Institutional Animal Ethics Committee (IAEC) of CSIR-IITR (#IITR/IAEC/47/14-17/2016). The animals were habituated for 1 week before the start of the experiment.

### Treatment schedule for two-stage skin carcinogenesis model

The dorsal surface of the mice was shaved with electrical clipper (Oster, WI, USA). 10 mice per group were randomized into 8 different groups, viz. Control, Vehicle control, SY (1%) alone, DMBA + TPA, DMBA + TPA + SY (0.1%), DMBA + TPA + SY (0.5%), DMBA + TPA + SY (1%), DMBA + TPA + 5-FU.

DMBA/TPA treatment protocol was adopted from an earlier study conducted in our laboratory with minor modification^22^. Instead of 120 nmol, only 40 nmol of DMBA in 200 µl of acetone was used for tumor initiation. This was done to mimic the environmental mutagen exposure scenario more closely by reducing the harshness of mutagen (DMBA) treatment. Briefly, the dorsal region of mice was shaved one day prior to any treatment involving DMBA or TPA. For tumor initiation, a topical application of 40 nmol of DMBA in 200 µl of acetone was administered one week prior to TPA and SY treatment.

For the subsequent tumor promotion stage, 4 nmol of TPA was applied twice a week topically, starting from the day following SY treatment, at the same site where DMBA was initially applied. This TPA treatment continued for a period of 21 successive weeks. To evaluate the potential chemopreventive effect, different doses of SY dissolved in DMSO (0.1%, 0.5%, and 1%) were topically applied the day before each TPA administration, three times a week, for the entire duration of 21 weeks. 5-Fluorouracil (5-FU) was used as a benchmark anti-cancer agent during the study. During the experimental tenure, the size of tumors larger than 1 mm was measured and recorded on a weekly basis. Additionally, the body weight of the mice was monitored weekly to assess any potential effects of the treatments on overall health. This experimental design allowed for the assessment of the chemopreventive effect of SY on skin tumor development in mice. The animals were euthanized at the end of the study period, using an overdose of thiopentone sodium (200 mg/kg, i.p.).

### In silico profiling of SY targets

In silico profiling of SY biological targets was carried out using the ‘Ligand Profiler’ protocol available in the Biovia Discovery Studio 2022 (DS2022), which maps the molecule to a set of pharmacophores present in the PharmaDB. The PharmaDB database included in the DS2022 contains over 250,000 pharmacophores models derived from 16,304 entries from the 2017 release of the sc-PDB protein data bank (http://bioinfo-pharma.u-strasbg.fr/scPDB). All the protein-ligand RCSB PDB structures flagged as targets by the pharmacophore screening of SY were filtered for human and murine models, followed by the ‘FitValue’ score, which indicates the goodness of fit of the ligand in the target binding cavity. For the top 10 target proteins based on the FitValue score, the binding affinity of SY was compared with the experimentally validated ligands on which the initial pharmacophore models were constructed. For this, the molecular docking analyses were performed using the ‘CDOCKER’ protocol available in the DS2022. For each comparison, a total of 10 random confirmations of SY and experimentally known ligands were generated using high-temperature molecular dynamics, which were then translated into the active sites of the proteins. Candidate poses were then created using random rigid-body rotations followed by simulated annealing. Afterward, a final minimization is used to refine the ligand poses in the binding cavity. For each target protein, the best binding affinity pose of SY and the corresponding ligand based on the CDOCKER_Energy score value were plotted on a swarm-plot using the Seaborn Python module. Lastly, a final plot was made showing the normalized rate of protein structures flagged as hits by screening over the total number of structures available on scPDB database for each protein. The structures were grouped by common UniProt ID, and the plot was made using the ggplot2 R library. The scPDB v.2017 database version was used for counting the number of structures available for each protein.

### Statical analysis

Statistical analysis was performed using GraphPad Prism software. The normally distributed data were analyzed using 1-way ANOVA followed by the Tukey post hoc test. The non-normally distributed data were compared using the Kruskal-Wallis test and Dunn’s multiple comparison test. The results were expressed as the mean ± SEM, and the *P*-value of less than 0.05 was considered significant.

### Reporting summary

Further information on research design is available in the Nature Research Reporting Summary linked to this article.

### Supplementary information

Supplementry Material

Supplementary Table 1

Supplementary Table 2

Reporting summary

## Data Availability

All data presented and/or analyzed in the current study are included in this manuscript and the Supplementary Files.
