# Supplementary material for: Sunset Yellow protects against oxidative damage and exhibits chemoprevention in chemically induced skin cancer model
Source: NPJ Syst Biol Appl. 2024 Mar 2;10:23. doi: 10.1038/s41540-024-00349-1 (PMC10908785; doi:10.1038/s41540-024-00349-1)
Supplement: Supplementary file 1 — Supplementry Material [file 41540_2024_349_MOESM1_ESM.pdf]

Supplementary Materials

Sunset Yellow, a food additive, protects against oxidative damage and exhibits chemopreventive efficacy in a model of chemically-induced skin cancer

Saurabh Singh[1,2], Sarika Yadav[1,2], Celine Cavallo[3,4], Durgesh Mourya[2,5], Ishu Singh[1,2], Vijay Kumar[1,2], Sachin Shukla[1,2], Pallavi Shukla[6], Romil Chaudhary[7], Gyan Prakash Maurya[7], Ronja Lea Jennifer Müller[4], Lilly Rohde[4], Aradhana Mishra[6], Olaf Wolkenhauer[4,8,9], Shailendra Gupta[4,9$*], Anurag Tripathi[1,2$*]

[1]Food Toxicology Group, CSIR- Indian Institute of Toxicology Research, 226001 Lucknow, India

[2]Academy of Scientific and Innovative Research (AcSIR), 201002 Ghaziabad, India

[3]University of Strasbourg, F-67081 Strasbourg, France

[4]Department of Systems Biology and Bioinformatics, University of Rostock, 18055 Rostock, Germany

[5]Drug and chemical toxicology group (FEST), CSIR- Indian Institute of Toxicology Research, 226001 Lucknow, India

[6]Division of Microbial Technology, CSIR-National Botanical Research Institute, 226001 Lucknow, India

[7]Center for Advanced Studies, Dr APJ Abdul Kalam Technical University, 226031 Lucknow, India

[8]Leibniz-Institute for Food Systems Biology at the Technical University of Munich, 85354 Freising, Germany

[9]Chhattisgarh Swami Vivekananda Technical University, 491107 Bhilai, India

[$]Equal senior author

*Corresponding author

anuragtripathi@iitr.res.in (Anurag Tripathi),

shailendra.gupta@uni-rostock.de (Shailendra Gupta)

Content Synopsis: Supplementary Figure 1-4

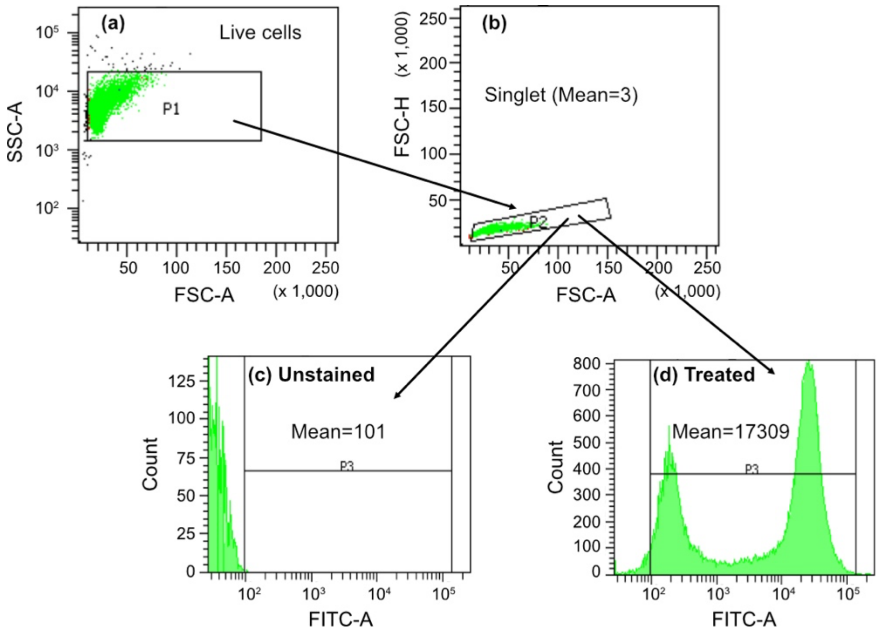

Supplementary Figure 1- Gating strategy based on FSC, SSC, Autofluorescence (Unstained) and Fluorescence; **(a)** Plot a (FSC-A versus SSC-A)—discrimination based on size (FSC) and granularity (SSC), the gate is used for calibration of the experiment **(Gate P1)** (dot plot); **(b) plot b** was used to exclude cells deviating from the main cohort of single cells (dot plot) **(Gate P2);** (c, d) **Plot c** depicts autofluorescence of unstained cells, used to set the threshold fluorescence of stained samples; **Plot d** showing cell population with high ROS levels denoted by DCFH-DA fluorescence **(Gate P3)** (log histogram).

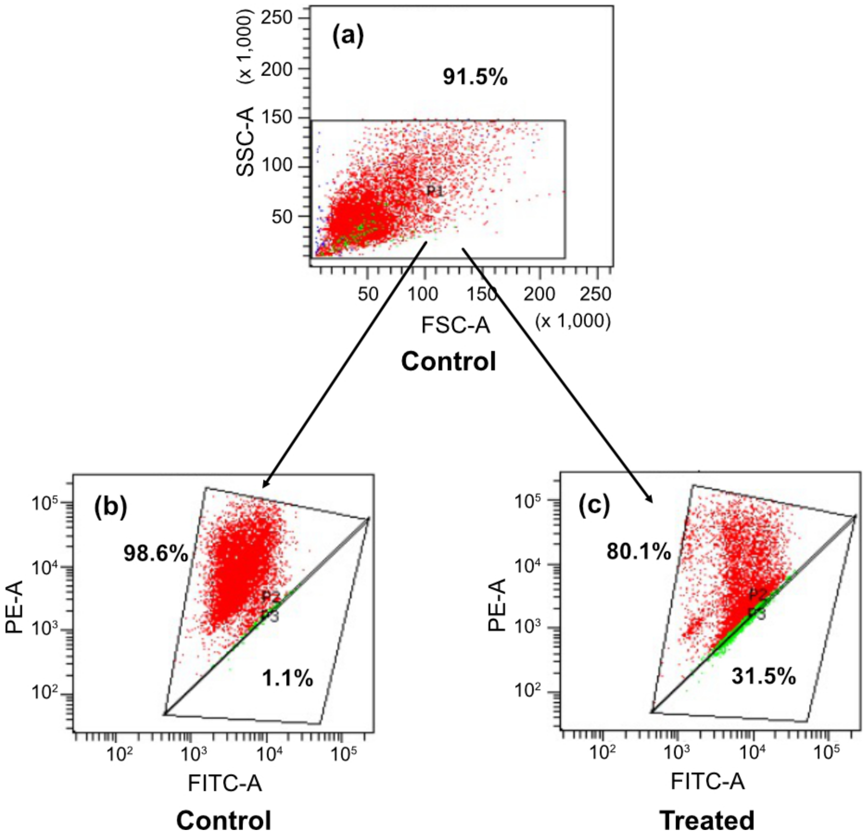

Supplementary Figure 2. Gating strategy based on FSC, SSC and JC-1 fluorescence. **(a)** Plot A (FSC-A versus SSC-A)—discrimination based on size (FSC) and granularity (SSC), the gate is used for calibration of the experiment; **(Gate p1)** (dot plot). **Plot (b, c)** (PE-A versus FITC-A)—**Gate P2 gate** represents cells with high MMP depicted in Red, and **Gate P3** represents cells with low MMP depicted in green (dot plot).

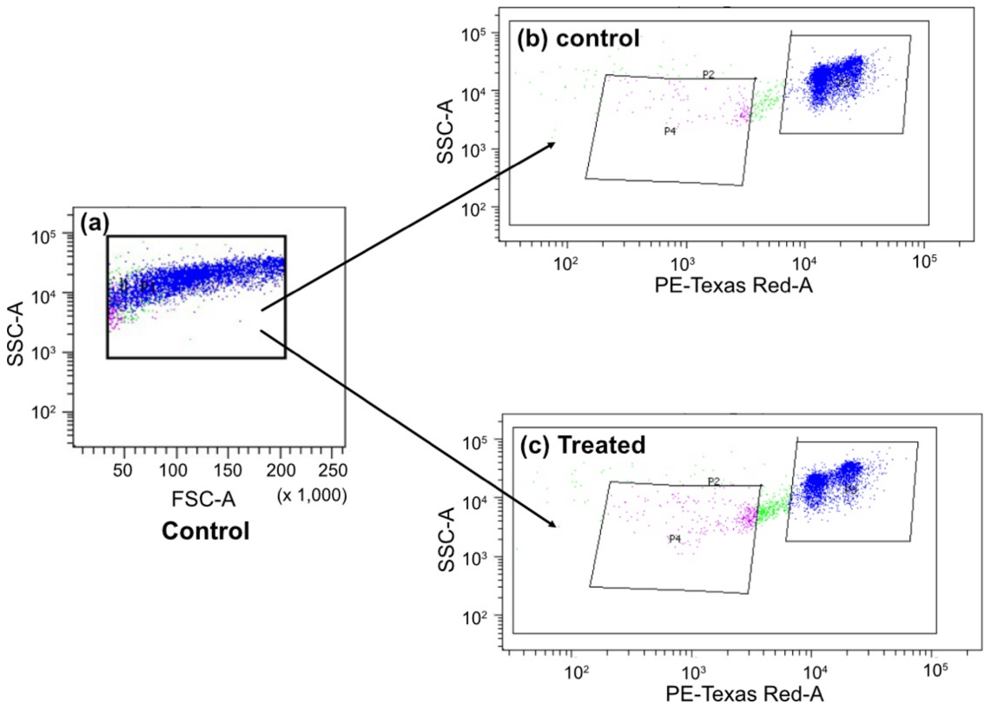

Supplementary Figure 3. Gating strategy based on FSC, SSC, PE-Texas Red A fluorescence. **Plot a** (FSC-A versus SSC-A)—discrimination based on size (FSC) and granularity (SSC), the gate is used for calibration of the experiment; **Plot (b, c)** Dot plot (PE-Texas Red-A versus SSC-A)—**Gate P4** represent MN formation with low granularity and fragmented DNA content, **Gate P3** depicts cells with intact nuclei. MNs (**Gate P3**) were defined as events between 1/100th and 1/ 10th of PE-Texas Red-A associated fluorescence (**Gate P4**).

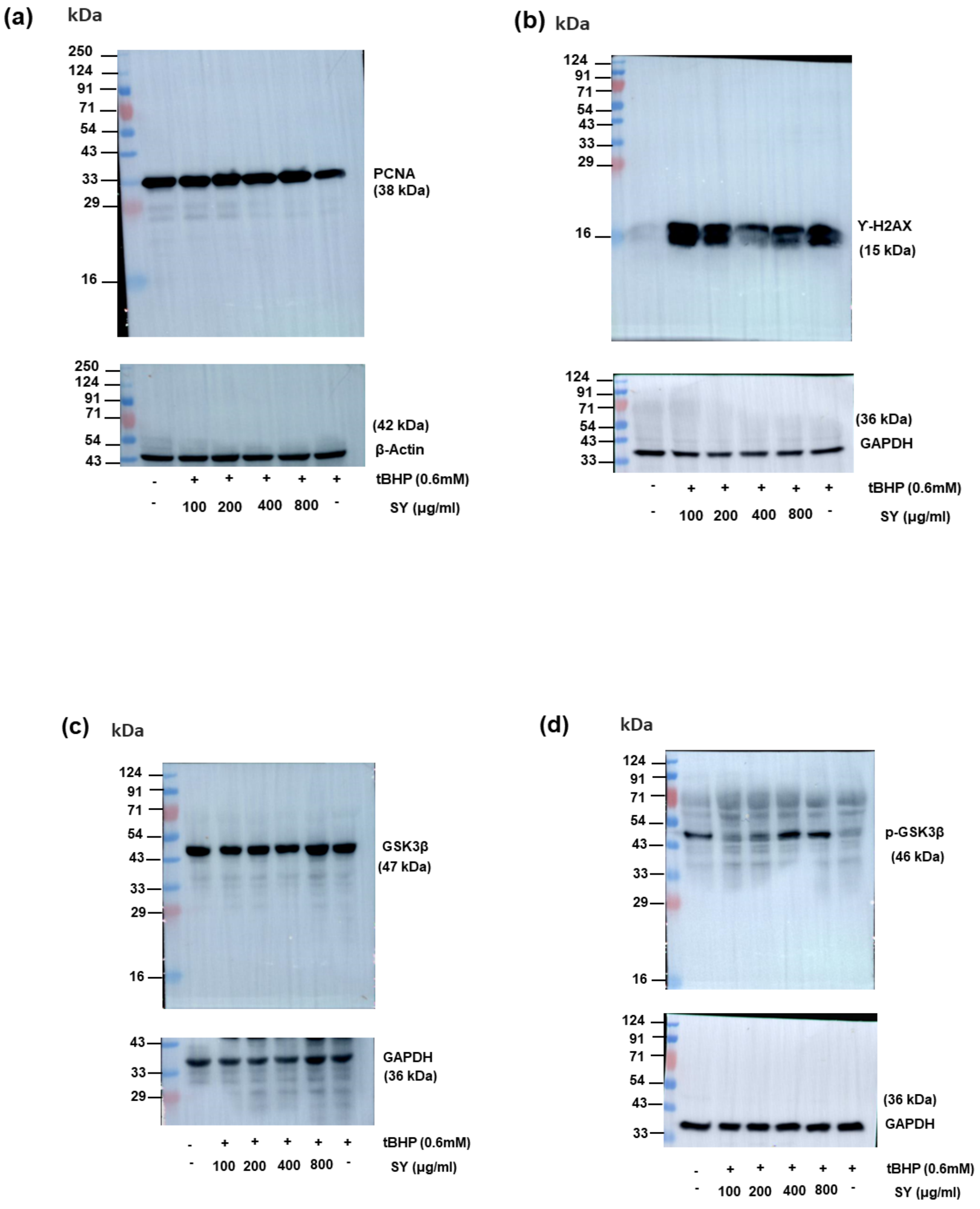

(a)
kDa
250
124
91
71
54
43
33
29
16
PCNA
(38 kDa)
(42 kDa)
β-Actin
tBHP (0.6mM)
SY (μg/ml)
100 200 400 800
(b)
kDa
124
91
71
54
43
33
29
16
Y-H2AX
(15 kDa)
(36 kDa)
GAPDH
tBHP (0.6mM)
SY (μg/ml)
100 200 400 800
(c)
kDa
GSK3β
(47 kDa)
GAPDH
(36 kDa)
tBHP (0.6mM)
SY (μg/ml)
100 200 400 800
(d)
kDa
p-GSK3β
(46 kDa)
(36 kDa)
GAPDH
tBHP (0.6mM)
SY (μg/ml)
100 200 400 800

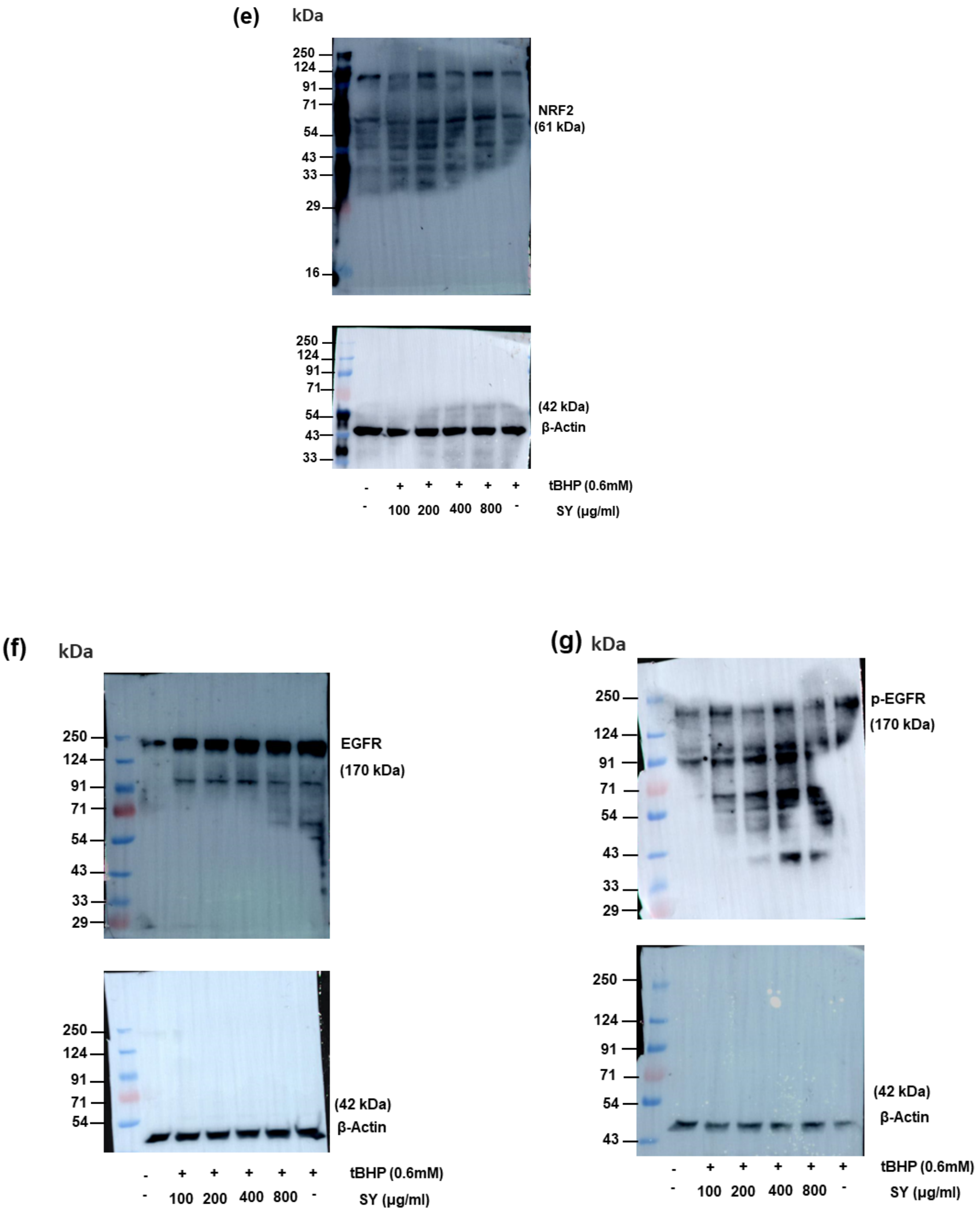

Supplementary Figure 4. Full lengths western blots: Effect of pretreating HaCaT cells with varying concentrations of SY (100-800µg/ml) on tBHP (0.6mM)-induced expression of different proteins. **Blot a** depicts a dose dependent enhancement in expression of PCNA protein; **Blot b** depicts a dose dependent decrease in the expression of γ-H2AX; **Blot c, d** depicts no change in expression of GSK3β expression but dose-dependent enhancement of phosphorylated GSK3β; **Blot e** depicts that SY pretreatment suppresses tBHP-induced NRF2 expression. **Blot f, g** depicts that pretreatment with SY didn't show any change in EGF-induced EGFR expression but notably suppressed EGF-induced p-EGFR expression.
