# Supplementary material for: Sunset Yellow protects against oxidative damage and exhibits chemoprevention in chemically induced skin cancer model
Source: NPJ Syst Biol Appl. 2024 Mar 2;10:23. doi: 10.1038/s41540-024-00349-1 (PMC10908785; doi:10.1038/s41540-024-00349-1)
Supplement: Supplementary file 4 — Reporting summary [file 41540_2024_349_MOESM4_ESM.pdf]

nature portfolio

Corresponding author(s): Shailendra Kumar Gupta, Anurag Tripathi

Last updated by author(s): Dec 14, 2023

Reporting Summary

Nature Portfolio wishes to improve the reproducibility of the work that we publish. This form provides structure for consistency and transparency in reporting. For further information on Nature Portfolio policies, see our Editorial Policies and the Editorial Policy Checklist.

Statistics

For all statistical analyses, confirm that the following items are present in the figure legend, table legend, main text, or Methods section.

n/a	Confirmed	
☐	☒	The exact sample size (*n*) for each experimental group/condition, given as a discrete number and unit of measurement
☐	☒	A statement on whether measurements were taken from distinct samples or whether the same sample was measured repeatedly
☐	☒	The statistical test(s) used AND whether they are one- or two-sided<br>*Only common tests should be described solely by name; describe more complex techniques in the Methods section.*
☐	☒	A description of all covariates tested
☐	☒	A description of any assumptions or corrections, such as tests of normality and adjustment for multiple comparisons
☐	☒	A full description of the statistical parameters including central tendency (e.g. means) or other basic estimates (e.g. regression coefficient) AND variation (e.g. standard deviation) or associated estimates of uncertainty (e.g. confidence intervals)
☐	☒	For null hypothesis testing, the test statistic (e.g. *F*, *t*, *r*) with confidence intervals, effect sizes, degrees of freedom and *P* value noted<br>*Give P values as exact values whenever suitable.*
☒	☐	For Bayesian analysis, information on the choice of priors and Markov chain Monte Carlo settings
☒	☐	For hierarchical and complex designs, identification of the appropriate level for tests and full reporting of outcomes
☒	☐	Estimates of effect sizes (e.g. Cohen's *d*, Pearson's *r*), indicating how they were calculated

Our web collection on statistics for biologists contains articles on many of the points above.

Software and code

Policy information about availability of computer code

Data collection	scPDB v. 2017 database (http://bioinfo-pharma.u-strasbg.fr/scPDB/) was used for pharmacophore-based screening.
Data analysis	Statistical analysis was performed using GraphPad Prism Software. Diagram and plots in Figure 7, and 9 are prepared using ggplot2 R library. In Figure 8, the CDOCKER_Energy scores were plotted on a swarm-plot using the Seaborn Python module. In silico profiling of SY was carried out using Biovia Discovery Studio 2022

For manuscripts utilizing custom algorithms or software that are central to the research but not yet described in published literature, software must be made available to editors and reviewers. We strongly encourage code deposition in a community repository (e.g. GitHub). See the Nature Portfolio guidelines for submitting code & software for further information.

Data

Policy information about availability of data

All manuscripts must include a data availability statement. This statement should provide the following information, where applicable:

- Accession codes, unique identifiers, or web links for publicly available datasets
- A description of any restrictions on data availability
- For clinical datasets or third party data, please ensure that the statement adheres to our policy

All data presented and/or analyzed in the current study are included in this manuscript and the supplementary files.

Research involving human participants, their data, or biological material

Policy information about studies with human participants or human data. See also policy information about sex, gender (identity/presentation), and sexual orientation and race, ethnicity and racism.

Reporting on sex and gender	*Use the terms sex (biological attribute) and gender (shaped by social and cultural circumstances) carefully in order to avoid confusing both terms. Indicate if findings apply to only one sex or gender; describe whether sex and gender were considered in study design; whether sex and/or gender was determined based on self-reporting or assigned and methods used. Provide in the source data disaggregated sex and gender data, where this information has been collected, and if consent has been obtained for sharing of individual-level data; provide overall numbers in this Reporting Summary. Please state if this information has not been collected. Report sex- and gender-based analyses where performed, justify reasons for lack of sex- and gender-based analysis.*
Reporting on race, ethnicity, or other socially relevant groupings	*Please specify the socially constructed or socially relevant categorization variable(s) used in your manuscript and explain why they were used. Please note that such variables should not be used as proxies for other socially constructed/relevant variables (for example, race or ethnicity should not be used as a proxy for socioeconomic status). Provide clear definitions of the relevant terms used, how they were provided (by the participants/respondents, the researchers, or third parties), and the method(s) used to classify people into the different categories (e.g. self-report, census or administrative data, social media data, etc.) Please provide details about how you controlled for confounding variables in your analyses.*
Population characteristics	*Describe the covariate-relevant population characteristics of the human research participants (e.g. age, genotypic information, past and current diagnosis and treatment categories). If you filled out the behavioural & social sciences study design questions and have nothing to add here, write "See above."*
Recruitment	*Describe how participants were recruited. Outline any potential self-selection bias or other biases that may be present and how these are likely to impact results.*
Ethics oversight	*Identify the organization(s) that approved the study protocol.*

Note that full information on the approval of the study protocol must also be provided in the manuscript.

Field-specific reporting

Please select the one below that is the best fit for your research. If you are not sure, read the appropriate sections before making your selection.

☒ Life sciences ☐ Behavioural & social sciences ☐ Ecological, evolutionary & environmental sciences

For a reference copy of the document with all sections, see nature.com/documents/nr-reporting-summary-flat.pdf

Life sciences study design

All studies must disclose on these points even when the disclosure is negative.

Sample size	n=10 for animal experiments and at least two technical replicates were taken for each in vitro experiment data point.
Data exclusions	No data was excluded from analysis.
Replication	Three independent experiments were carried out for each in vitro study.
Randomization	Randomization of animals in all the experimental groups was carried out without any bias.
Blinding	All the animals and sample vials/wells were coded and the experimenters taking the observations were blind to the study groups and treatments.

Behavioural & social sciences study design

All studies must disclose on these points even when the disclosure is negative.

Study description	*Briefly describe the study type including whether data are quantitative, qualitative, or mixed-methods (e.g. qualitative cross-sectional, quantitative experimental, mixed-methods case study).*
Research sample	*State the research sample (e.g. Harvard university undergraduates, villagers in rural India) and provide relevant demographic information (e.g. age, sex) and indicate whether the sample is representative. Provide a rationale for the study sample chosen. For studies involving existing datasets, please describe the dataset and source.*
Sampling strategy	*Describe the sampling procedure (e.g. random, snowball, stratified, convenience). Describe the statistical methods that were used to predetermine sample size OR if no sample-size calculation was performed, describe how sample sizes were chosen and provide a*

	rationale for why these sample sizes are sufficient. For qualitative data, please indicate whether data saturation was considered, and what criteria were used to decide that no further sampling was needed.
Data collection	*Provide details about the data collection procedure, including the instruments or devices used to record the data (e.g. pen and paper, computer, eye tracker, video or audio equipment) whether anyone was present besides the participant(s) and the researcher, and whether the researcher was blind to experimental condition and/or the study hypothesis during data collection.*
Timing	*Indicate the start and stop dates of data collection. If there is a gap between collection periods, state the dates for each sample cohort.*
Data exclusions	*If no data were excluded from the analyses, state so OR if data were excluded, provide the exact number of exclusions and the rationale behind them, indicating whether exclusion criteria were pre-established.*
Non-participation	*State how many participants dropped out/declined participation and the reason(s) given OR provide response rate OR state that no participants dropped out/declined participation.*
Randomization	*If participants were not allocated into experimental groups, state so OR describe how participants were allocated to groups, and if allocation was not random, describe how covariates were controlled.*

Ecological, evolutionary & environmental sciences study design

All studies must disclose on these points even when the disclosure is negative.

Study description	*Briefly describe the study. For quantitative data include treatment factors and interactions, design structure (e.g. factorial, nested, hierarchical), nature and number of experimental units and replicates.*
Research sample	*Describe the research sample (e.g. a group of tagged Passer domesticus, all Stenocereus thurberi within Organ Pipe Cactus National Monument), and provide a rationale for the sample choice. When relevant, describe the organism taxa, source, sex, age range and any manipulations. State what population the sample is meant to represent when applicable. For studies involving existing datasets, describe the data and its source.*
Sampling strategy	*Note the sampling procedure. Describe the statistical methods that were used to predetermine sample size OR if no sample-size calculation was performed, describe how sample sizes were chosen and provide a rationale for why these sample sizes are sufficient.*
Data collection	*Describe the data collection procedure, including who recorded the data and how.*
Timing and spatial scale	*Indicate the start and stop dates of data collection, noting the frequency and periodicity of sampling and providing a rationale for these choices. If there is a gap between collection periods, state the dates for each sample cohort. Specify the spatial scale from which the data are taken*
Data exclusions	*If no data were excluded from the analyses, state so OR if data were excluded, describe the exclusions and the rationale behind them, indicating whether exclusion criteria were pre-established.*
Reproducibility	*Describe the measures taken to verify the reproducibility of experimental findings. For each experiment, note whether any attempts to repeat the experiment failed OR state that all attempts to repeat the experiment were successful.*
Randomization	*Describe how samples/organisms/participants were allocated into groups. If allocation was not random, describe how covariates were controlled. If this is not relevant to your study, explain why.*
Blinding	*Describe the extent of blinding used during data acquisition and analysis. If blinding was not possible, describe why OR explain why blinding was not relevant to your study.*

Did the study involve field work? ☐ Yes ☐ No

Field work, collection and transport

Field conditions	*Describe the study conditions for field work, providing relevant parameters (e.g. temperature, rainfall).*
Location	*State the location of the sampling or experiment, providing relevant parameters (e.g. latitude and longitude, elevation, water depth).*
Access & import/export	*Describe the efforts you have made to access habitats and to collect and import/export your samples in a responsible manner and in compliance with local, national and international laws, noting any permits that were obtained (give the name of the issuing authority, the date of issue, and any identifying information).*
Disturbance	*Describe any disturbance caused by the study and how it was minimized.*

Reporting for specific materials, systems and methods

We require information from authors about some types of materials, experimental systems and methods used in many studies. Here, indicate whether each material, system or method listed is relevant to your study. If you are not sure if a list item applies to your research, read the appropriate section before selecting a response.

Materials & experimental systems

n/a	Involved in the study
☐	☒ Antibodies
☐	☒ Eukaryotic cell lines
☒	☐ Palaeontology and archaeology
☐	☒ Animals and other organisms
☒	☐ Clinical data
☒	☐ Dual use research of concern
☒	☐ Plants

Methods

n/a	Involved in the study
☒	☐ ChIP-seq
☐	☒ Flow cytometry
☒	☐ MRI-based neuroimaging

Antibodies

Antibodies used

Antibodies used PCNA mouse monoclonal antibody Lot: 0427729-1 (Clone IPO-38, Cat#10004805, were obtained from Cayman Chemical, Ann Arbor, MI applicable for western blot, GAPDH (sc-137179) (Lot: 12717) mouse monoclonal IgG1 applicable for western blot, β-actin (sc-47778) (Lot-K1418), Phospho-GSK3β (Ser9) (sc-11757) Lot #L0414 rabbit polyclonal IgG and NRF2 rabbit polyclonal IgG (Sc13032) (Lot D0815) from Santa Cruz Biotechnology (Santa Cruz, CA, USA). Phospho-Histone H2A.X (D7T2V) Lot: 1 Mouse mAb #80312, antibody applicable for western blot, Antibodies against EGFR (#2232S) (Lot: 15) Rabbit, p-EGFR (#2235S) Rabbit (Lot: 7) were purchased from Cell Signaling Technology (Beverly, MA, USA), while anti-GSK3β (#610202), Lot:5089584 was obtained from BD Biosciences. Subsequently, secondary antibodies, Goat anti-mouse (#31430) and Goat anti-rabbit (#31460) were obtained from Invitrogen, USA.

Validation

Antibodies used PCNA mouse monoclonal antibody (+) multiple species PCNA (species and tissue-unspecific), were obtained from Cayman Chemical, Ann Arbor, MI applicable for western blot, GAPDH mouse monoclonal IgG1 GAPDH is recommended for detection of GAPDH of mouse, rat and human origin by Western Blotting, β-actin is a mouse monoclonal antibody raised against gizzard Actin of chicken origin and recommended for detection of β-Actin of mouse, rat, human, avian, bovine, canine, porcine, rabbit, Dictyostelium discoideum and Physarum polycephalum origin by Western Blotting, p-GSK-3β (Ser 9) is available as either goat (sc-11757) or rabbit (sc-11757-R) polyclonal affinity purified antibody and recommended for detection of Ser 9 phosphorylated GSK-3β of mouse, rat, human, Xenopus laevis and zebrafish origin by Western Blotting and NRF2 rabbit polyclonal antibody raised against amino acids 37-336 of Nrf2 of human origin and Nrf2 (H-300) is recommended for detection of Nrf2 of mouse, rat and human origin by Western Blotting from Santa Cruz Biotechnology (Santa Cruz, CA, USA). Phospho-Histone H2A.X (D7T2V) Phospho-Histone H2A.X (Ser139) (D7T2V) Mouse mAb recognizes endogenous levels of Histone H2A.X protein only when phosphorylated at Ser139 applicable for western blot, Antibodies against EGFR (#2232S) (Lot: 15) Polyclonal antibodies are produced by immunizing animals with a synthetic peptide corresponding to residues surrounding Tyr1068 of human EGF receptor. Antibodies are purified by protein A and peptide affinity chromatography. Species reactivity is determined by testing in at least one approved application (e.g., western blot)., p-EGFR Phospho-EGF Receptor (Tyr992) Antibody detects endogenous EGF receptor only when phosphorylated at tyrosine 992. The antibody may cross-react with other activated EGF receptor family members (e.g. ErbB2), Species Reactivity:Human, Mouse, Monkey applicable for western blot were purchased from Cell Signaling Technology (Beverly, MA, USA), while anti-GSK3β Reactivity: Canis lupus familiaris (Domestic dog), Gallus gallus domesticus (Chicken), Homo sapiens (Human), Mus musculus (House mouse), Rattus norvegicus (Rat) applicable for western blot was obtained from BD Biosciences. Subsequently, secondary antibodies, Goat anti-mouse has been successfully used in Western blot, IHC and IP applications. Product # 31430 reacts with the heavy chains of mouse igg and with the light chains common to most mouse immunoglobulins applicable for western blot, and Goat anti-rabbit (A peroxidase (HRP)-conjugated polyclonal goat anti-rabbit antibody, applicatios used include western blot, were obtained from Invitrogen, USA.

Eukaryotic cell lines

Policy information about cell lines and Sex and Gender in Research

Cell line source(s)

Origin: In vitro spontaneously transformed keratinocytes from histologically normal skin.
Species: Homo sapiens

Authentication

Catalog #: T0020001, Lot Number: 0003798; Authenticity of cell line was stabilised by STR analysis.

Mycoplasma contamination

Not detected

Commonly misidentified lines
(See ICLAC register)

None

Animals and other research organisms

Policy information about studies involving animals; ARRIVE guidelines recommended for reporting animal research, and Sex and Gender in Research

Laboratory animals	BALB/c female mice (6-7 weeks)
Wild animals	No
Reporting on sex	Sex based analysis was not performed. It is well established that the skin carcinogenesis model used in present study holds equally good for both sex.
Field-collected samples	n/a
Ethics oversight	For all animal experiments permission were granted by the Institutional Animal Ethics Committee (IAEC) of CSIR-IITR (#IITR/IAEC/47/14-17/2016).

Note that full information on the approval of the study protocol must also be provided in the manuscript.

Plants

Seed stocks	*Report on the source of all seed stocks or other plant material used. If applicable, state the seed stock centre and catalogue number. If plant specimens were collected from the field, describe the collection location, date and sampling procedures.*
Novel plant genotypes	*Describe the methods by which all novel plant genotypes were produced. This includes those generated by transgenic approaches, gene editing, chemical/radiation-based mutagenesis and hybridization. For transgenic lines, describe the transformation method, the number of independent lines analyzed and the generation upon which experiments were performed. For gene-edited lines, describe the editor used, the endogenous sequence targeted for editing, the targeting guide RNA sequence (if applicable) and how the editor was applied.*
Authentication	*Describe any authentication procedures for each seed stock used or novel genotype generated. Describe any experiments used to assess the effect of a mutation and, where applicable, how potential secondary effects (e.g. second site T-DNA insertions, mosiacism, off-target gene editing) were examined.*

Flow Cytometry

Plots

Confirm that:

☒ The axis labels state the marker and fluorochrome used (e.g. CD4-FITC).

☒ The axis scales are clearly visible. Include numbers along axes only for bottom left plot of group (a 'group' is an analysis of identical markers).

☒ All plots are contour plots with outliers or pseudocolor plots.

☒ A numerical value for number of cells or percentage (with statistics) is provided.

Methodology

Sample preparation	The sample preparation for different flowcytometry based assays has been elaborated in the "Material and Methods" section.
Instrument	FACS Canto II, BD Biosciences, San Jose, CA
Software	BD FACSDiva™ Software
Cell population abundance	1 × 10^6 cells
Gating strategy	Gating strategy was based on FSC, SSC, Autofluorescence (Unstained) and Fluorescence; (FSC-A versus SSC-A)—discrimination based on size (FSC) and granularity (SSC), the gate is used for calibration of the experiment (Gate P1) (dot plot). After that we excluded cells deviating from the main cohort of single cells (dot plot) (Gate P2). Then autofluorescence of unstained cells was used to set the threshold fluorescence of stained samples. Detailed strategy and the plots have been provided in the supplementary files.

☒ Tick this box to confirm that a figure exemplifying the gating strategy is provided in the Supplementary Information.
